# Supplementary material for: Allosteric regulation and crystallographic fragment screening of SARS-CoV-2 NSP15 endoribonuclease
Source: Nucleic Acids Res. 2023 Apr 28;51(10):5255–70. doi: 10.1093/nar/gkad314 (PMC10250223; doi:10.1093/nar/gkad314)
Supplement: gkad314_Supplemental_Files [file gkad314_supplemental_files.zip › Supplementary datasets.zip!/160/Refine_8/01-BUSTER/Cycle-2/LIST.html]

Refining

**BUSTER 2.X**: A suite of programs for macromolecular structure refinement by maximum-likelihood and Bayesian methods  
Copyright © 1992-2020 Global Phasing Ltd.  
All rights reserved.  
Authors: G. Bricogne, E. Blanc, M. Brandl, C. Flensburg, P. Keller, W. Paciorek, P. Roversi, O. Smart, C. Vonrhein, T. Womack  

Please reference use of the software as:

- BUSTER-TNT 2.X, Global Phasing Ltd, Sheraton House, Cambridge CB3 0AX, UK

Version: 2.10.3 for linux64-ifort made on May 20 2020 at 01:14:34  
Licenced to: Diamond Light Source, Scientific Software  

```
---


### Parsing of the BUSTER cardfile

TITLE : autoBUSTER refinement

   Cell information

   Symmetry information

 Using CCP4 X-ray form factors.

   Missing structure chemical composition

 Using ATOM block for chemical composition.


   Shell information

### Parsing of the TNT cardfile


---


### Reading observations file

 Number of reflexions in input:  62372

      Number of accepted reflexions:  58271

      Number of rejected reflexions:      0

   Reflexions in test set:            2953

   Reflexions in working set:        55318


---


### 1. Generating initial model

 2.10 * Shannon sampling rate. NX NY NZ:   288  288  240


  Effective Shannon rates are:    2.22    2.22    2.55


   Allocated BUFFER of size:     76.465 Mb.

   Initial Geometry Information


 No missing atoms declared


   Summary of model volumes

 No missing atoms declared: no prior computed


 Computing bulk solvent model scattering


 NCSMASK PDB radius for solvent mask:     2.15 Angstrom

 running PDBSET (setting cell and spacegroup P1) ... done
 running NCSMASK [1] (creating mask, SMOOTH 1.04572) ... done
 running MAPDUMP (to check if reduction to unit cell is needed) ... done
 running MAPMASK (converting mask to map) ... done

   Generation of babslv by theta-filter

   Electron density in frg mask:  0.420 Å-1

 FOBS  put on rough absolute scale by Wilson plot :
           K        =  0.66940E+02
           B        =     -9.837

 

### Cycle 0001: MaxLik scaling and refinement


   MaxLik Scaling Cycle 0001

   Wilson plot

   Statistics in resolution bins just after scaling
```

|  |  |
| --- | --- |
|  | - Rxpct factors plot- Rxpct factors table- <Log-Lik Gain>- Ln Sigma A- Correlation Coefficients |

```
   <Log-Likelihood> of null-hypothesis

Structure Factor Amplitudes


   Average Figure of Merit

 Switching off refinement of overall temperature factor


 Added B =     0.3880 Å2 to all atoms in fragment


 Added B =     0.3880 Å2 to B_SOLV

```
gelly refinement module
 Authors (2004-2009) O. S. Smart, C. Flensburg, W. Paciorek, C. Vonrhein, 
                     M. Brandl, T. O. Womack and G. Bricogne
         (2010-2011) O. S. Smart, C. Flensburg, W. Paciorek, C. Vonrhein, 
                     T. O. Womack and G. Bricogne
         (2012-2014) O. S. Smart, C. Flensburg, W. Paciorek, C. Vonrhein, 
                     and G. Bricogne
         (2015-2020) G. Bricogne, C. Flensburg, W. Paciorek, C. Vonrhein
 Copyright © 2004-2020 Global Phasing Ltd. 
 All rights reserved.
 gelly module compilation date May 20 2020 01:11:20
 
 gelly uses routines from TNT geometry. References: 
     Tronrud, D. E., Ten Eyck, L. F., Matthews, B. W. (1987)
     "An Efficient General-Purpose Least-Squares Refinement Program 
     for Macromolecular Structures". Acta Crystallogr A, 43:489-501.
     Tronrud, D. E. (1997). "The TNT Refinement Package", 
     in Macromolecular Crystallography, Part B, Eds Charlie Carter 
     and Robert Sweet, Methods in Enzymology, 277:306-319.
 wildcard matching function by Julian Robichaux http://www.nsftools.com

 No -random_seed flag found. Random number stream initialised using:
2147483562
2147483398

 Control flags set to:  -autoncs -hydrogens_input_occ2zero \
                     -hydrogens_output_occ2heavy -screen \
                     100 -screen_sigma \
                     3.0 -keeppdb \
                     1000000 -taomonitor_verbose \
                     2 
 

 ############### Starting setup via TNT routines

 Setting up tnt routines including license check:

                   TNT Stereochemistry Program
 Version: 5.22.3.0
 Licence for Diamond Light Source, Scientific Software is valid
 
 Using TNT geometry routines to load the tnt control file: gelly.tnt
 Using TNT routines to load initial coordinates from file fragment.cor
 Expecting 6503 atoms

 Found     2188 "NOTE BUSTER_RESIDUE_ATOM_DATA" cards
 Have copied information for    6503 atoms from TNT to gelly's store 
 
 The TNT WEIGHT cards used by this run:
 -----WEIGHT       BOND     2.0
 -----WEIGHT       ANGLE    2.0
 -----WEIGHT       IMPROPER 0.0
 -----WEIGHT       TORSION  2.0
 -----WEIGHT       PSEUDO   0.0
 -----WEIGHT       TRIGONAL 2.0
 -----WEIGHT       PLANE    5.0
 -----WEIGHT       CONTACT  5.0
 -----WEIGHT       BCORREL  10.0
 -----WEIGHT       CHIRAL   5.0
 -----WEIGHT       NCS      50.0
 -----WEIGHT       IDEAL    4.0
 -----WEIGHT       RFACTOR  7.45
 
 Dictionary versions picked up from NOTE BUSTER_RESTRAINT_DICT cards as: 
 ------ protgeo_eh99.dat (V1.6.4.17) 20191001  STANDARD AMINO ACID DICTIONARY. BONDS AND ANGLES FROM ENGH AND HUBER EH99. OTHER VALUES BASED ON PREVIOUS TNT OR TAKEN FROM CCP4. INCLUDES HYDROGEN ATOMS.
 ------ exoticaa.dat (V1.3.4.15) 20200327  COLLECTION OF NON-STANDARD AMINO ACIDS, MAINLY EH91 WITHOUT IDEAL DISTANCE INFO
 ------ nuclgeo.dat (V1.13.4.8) 20190909
 ------ bcorrel.dat (V1.15.10.2) 20190616
 ------ contact.dat (V1.15.12.10) 20191121
 ------ idealdist_contact.dat (V1.3.4.12) 20191001  IDEAL-DISTANCE CONTACT TERM DATA AS USED IN PROLSQ. VALUES USED HERE ARE BASED ON DATA HARVESTING OF PDB AS OF 20190109.
 ------ restraints for LIG (Unknown) from cif dictionary Z1636723439.cif
 ------ restraints for CIT (CITRIC ACID) from cif dictionary CIT.cif; generated by GRADE_PDB_LIGAND 1.2.12 (October 4 2016) using MOGUL 1.7.2(RC1), CSD as538be, with quantum mechanics RM1 ((DatabaseID PDB-CIT))
 ------ assume.dat (V1.9.4.2) 20180705
 
 Have picked up spacegroup name for pdb output as 'P 63      '
 Setting up Chain/Residue arrays
 
 Have command line specification -autoncs*
     Have found       1 NCS relations
     Pairwise matching residue number and type between chains: 
                    A        B        S        W    
          A         .       99%       -        -   
          B                  .        -        -   
          S                           .        -   
          W                                    .   
       Table key--                                                       
          all   : two chains have 100% match and same number of residues 
         multi  : ditto and they contribute to a multi chain ncs cluster 
          ***%  : two chains have >80% matching residue number/type      
           -    : chains appear to be unrelated                          
      chain A     have identified ncs to     1 other chains. So will weight each autoncs by (       6.666/#)=      6.6660
      chain B     have identified ncs to     1 other chains. So will weight each autoncs by (       6.666/#)=      6.6660
      chain S     have identified ncs to     0 other chains. 
      chain W     have identified ncs to     0 other chains. 
     For .Gelly cards produced see below - after pruning
 
 Setting up BUSTER_SET groups
 Have found    89 BUSTER_SET cards
 Now silently applying automatic/dictionary set definitions. For a verbose listing use '-verbose_set' flag.
 Applying user defined set definitions:
 =====NOTE BUSTER_SET ncsautoXcld = Water --> set 'ncsautoXcld' now has   966 atoms 
 =====NOTE BUSTER_SET ncsautoABset = Chain_A \ ncsautoXcld --> set 'ncsautoABset' now has  2770 atoms 
 Have defined    49 sets: 
      set:                 'All' has   6503 atoms in   1666 residues - automatically defined
      set:               'Empty' has      0 atoms in      0 residues - automatically defined
      set:            'ExoticAA' has      0 atoms in      0 residues - defined by dictionary file
      set:           'ExoticNUC' has      0 atoms in      0 residues - defined by dictionary file
      set:              'NotHET' has   5514 atoms in    698 residues - defined by dictionary file
      set:             'Chain_A' has   2770 atoms in    350 residues - automatically defined
      set:             'Chain_B' has   2767 atoms in    350 residues - automatically defined
      set:             'Chain_S' has    567 atoms in    567 residues - automatically defined
      set:             'Chain_W' has    399 atoms in    399 residues - automatically defined
      set:               'Water' has    966 atoms in    966 residues - defined by dictionary file
      set:          'StdProtein' has   5514 atoms in    698 residues - defined by dictionary file
      set:             'Protein' has   5514 atoms in    698 residues - defined by dictionary file
      set:                'Back' has   2794 atoms in    698 residues - defined by dictionary file
      set:                'Side' has   2720 atoms in    650 residues - defined by dictionary file
      set:        'RnaCanonical' has      0 atoms in      0 residues - defined by dictionary file
      set:        'DnaCanonical' has      0 atoms in      0 residues - defined by dictionary file
      set:     'RnaDnaCanonical' has      0 atoms in      0 residues - defined by dictionary file
      set:     'DnaRnaCanonical' has      0 atoms in      0 residues - defined by dictionary file
      set:         'RnaExtended' has      0 atoms in      0 residues - defined by dictionary file
      set:         'DnaExtended' has      0 atoms in      0 residues - defined by dictionary file
      set:      'RnaDnaExtended' has      0 atoms in      0 residues - defined by dictionary file
      set:      'DnaRnaExtended' has      0 atoms in      0 residues - defined by dictionary file
      set:                 'Dna' has      0 atoms in      0 residues - defined by dictionary file
      set:                 'Rna' has      0 atoms in      0 residues - defined by dictionary file
      set:              'RnaDna' has      0 atoms in      0 residues - defined by dictionary file
      set:              'DnaRna' has      0 atoms in      0 residues - defined by dictionary file
      set:             'Nucleic' has      0 atoms in      0 residues - defined by dictionary file
      set:             'Polymer' has   5514 atoms in    698 residues - defined by dictionary file
      set: 'BufferWeichenberger' has     13 atoms in      1 residues - defined by dictionary file
      set:    'BufferAdditional' has      0 atoms in      0 residues - defined by dictionary file
      set:              'Buffer' has     13 atoms in      1 residues - defined by dictionary file
      set:         'MetalAlkali' has      0 atoms in      0 residues - defined by dictionary file
      set:  'MetalAlkalineEarth' has      0 atoms in      0 residues - defined by dictionary file
      set:     'MetalTransition' has      0 atoms in      0 residues - defined by dictionary file
      set: 'MetalPostTransition' has      0 atoms in      0 residues - defined by dictionary file
      set:    'MetalLanthanides' has      0 atoms in      0 residues - defined by dictionary file
      set:               'Metal' has      0 atoms in      0 residues - defined by dictionary file
      set:         'SugarAlphaD' has      0 atoms in      0 residues - defined by dictionary file
      set:         'SugarAlphaL' has      0 atoms in      0 residues - defined by dictionary file
      set:               'Sugar' has      0 atoms in      0 residues - defined by dictionary file
      set:               'Other' has     23 atoms in      2 residues - defined by dictionary file
      set:            'Hydrogen' has      0 atoms in      0 residues - defined by dictionary file
      set:         'nonHydrogen' has   6503 atoms in   1666 residues - defined by dictionary file
      set:             'OccZero' has      0 atoms in      0 residues - defined by dictionary file
      set:          'OccNonZero' has   6503 atoms in   1666 residues - defined by dictionary file
      set:             'OccFull' has   6461 atoms in   1663 residues - defined by dictionary file
      set:          'OccNonFull' has     42 atoms in      3 residues - defined by dictionary file
      set:         'ncsautoXcld' has    966 atoms in    966 residues - user specified
      set:        'ncsautoABset' has   2770 atoms in    350 residues - user specified
 No command line arguments '-occ_allhet' or '-occ_restyp' so will not setup automated occcupancy refinement
 
 Interpreting CONSTANT and FREE cards: 
 =====CONSTANT     OCC
 Have found and interpreted      1 CONSTANT cards in the TNT input. 
 Before any COMBINE - Number of atoms with fixed XYZ =     0
 Before any COMBINE - Number of atoms with fixed B   =     0
 Before any COMBINE - Number of atoms with fixed OCC =  6503
 Before any COMBINE - Number of atoms with fixed BIJ =   966
                            cf total number of atoms =  6503
 
 There are no COMBINE cards to be found in the TNT card input 
 
 There are no NOTE BUSTER_COMBINE_RES cards to be found in the TNT card input 

 ############### Working out full connectivity list


 ############### Working out equivalent atoms


 Residue with most atoms given below:
        A|58:N(TRP)         
        A|58:CA(TRP)        
        A|58:CB(TRP)        
        A|58:CG(TRP)        
        A|58:CD1(TRP)       
        A|58:NE1(TRP)       
        A|58:CE2(TRP)       
        A|58:CD2(TRP)       
        A|58:CE3(TRP)       
        A|58:CZ3(TRP)       
        A|58:CH2(TRP)       
        A|58:CZ2(TRP)       
        A|58:C(TRP)         
        A|58:O(TRP)         


 Have no '-equiv_check_plus' argument - so will take atom type into
 account when checking for equivalence of otherwise identical atoms.
 

 ############### Looking at dihedrals


 ############### Looking at chirals

 NOTE BUSTER_CHIRAL_ANGLECUT card found - read value=    -31.00 degs from it
 NOTE BUSTER_CHIRAL_SIGMA card found - read value=      1.70 degs from it
 RANGE allowed for B variables in refinement:   from      3.00000 to    300.00000

 ############### Preparing for PDB output


 ############### Processing extra restraints explicitly defined

 No 'NOTE BUSTER_TARGET' cards or -target?? args found (used to read coordinates files for soft ncs to external)
 No 'NOTE BUSTER_OCCSUM'     cards found - so not using sum of occupancy restraints
 No 'NOTE BUSTER_DISTANCE'   cards found - so not setting up any additional atom-atom distance restraints
 No 'NOTE BUSTER_UTILANGLE'  cards found - so not setting up any additional restraints on any angle
 No 'NOTE BUSTER_UTILTOR'    cards found - so not setting up any additional sinusoidal restraints on torsions
 
 --- Have some similarity definitions 
     Similarity restraints on RMSD use superposition routines from: 
     Coutsias, E.A., Seok, C., Dill, K.A.(2004)
     "Using quaternions to calculate RMSD", J. Comput. Chem., 25:1849-1857 

 Have found    1 'NOTE BUSTER_SIM_DEFINE' cards 
     Maximum number of chains involved in any SIM group (excluding the template)=     1


 ########## Interpreting card: NOTE BUSTER_SIM_DEFINE ncsautoAB ncsautoABset B
     Number of atoms in template set=  2770
     Number of related chains (other than template) in SIM set=    1
     List of chains to be restrained (excluding template)=   B   
     Pairwise RMSD, XYZ in Angstroms, for SIM group: ncsautoAB
                 A         B   
     A         -----     ----- 
     B         0.599     ----- 
     Overall RMSD,XYZ for the SIM group is     0.599 Angstroms
     Pairwise rms deviation of [individual B's minus their set/chain average] in Angs**2
                 A         B         Bavg
     A         -----     -----     81.75
     B         23.39     -----     67.33
     Overall RMSD,B   for the SIM group is    23.394 Angs**2
     The number of common atoms for the above fits:
                 A         B   
     A         -----     ----- 
     B          2757     ----- 
     (compared to the template set that has    2770 atoms in it)
     List of atoms that are absent in one or more chains for this group: 
                                    A    B   
                    C1      401     +    -
                    O1      401     +    -
                    O2      401     +    -
                    C2      401     +    -
                    C3      401     +    -
                    O7      401     +    -
                    C4      401     +    -
                    C5      401     +    -
                    O3      401     +    -
                    O4      401     +    -
                    C6      401     +    -
                    O5      401     +    -
                    O6      401     +    -
     (key  +  atom present for this chain 
           -  atom absent  for this chain)
 transforms for each of the superpositions orthogonal matrix r11 r12 r13 r21 r22 r23 r31 r32 r33 tx ty tz (PDBSET syntax): 
TRANS_AB -0.877958 -0.478556 -0.013199 -0.478612  0.878026  0.001264  0.010985  0.007427 -0.999912    33.933    32.950    15.211
 reverse transforms 
TRANS_BA -0.877958 -0.478612  0.010985 -0.478556  0.878026  0.007427 -0.013199  0.001264 -0.999912    45.395   -12.805    15.616
 
 Setting up LSSR interatomic distance restraints
 Interpreting card: NOTE BUSTER_SIM_RESTRAIN_LSSR ncsautoAB 6.666
        setting up interatomic distance pair restraints with:
        Dmin=         0.00
        Dmax=         5.50
        sigma=        0.20
        Vmax=         3.00
        weight=       6.67
        number of distance pairs defined for this set is      34799
 Have not supplied either '-sim_swap_equiv' or '-sim_swap_equiv_plus' argument 
   so will not try swaping equivalent atoms to improve NCS and/or target agreement.
 
 NCS by LSSR outlier analysis
 -autoncs option automatic NCS prune routine -------- 
 Residues pruned out by gradient condition:
           A|34     A|135     A|137     A|147     A|148     A|150     A|151
          A|173     A|181     A|184     A|186     A|187     A|189     A|216
          A|217     A|220     A|234     A|235     A|236     A|238     A|239
          A|244     A|245     A|246     A|247     A|248     A|249     A|250
          A|253     A|256     A|280     A|281     A|287     A|288     A|289
          A|292     A|293     A|294     A|295     A|318     A|319     A|327
          A|328     A|329     A|343     A|344     A|345     A|346      B|34
          B|135     B|137     B|147     B|148     B|150     B|151     B|173
          B|181     B|184     B|186     B|187     B|189     B|216     B|217
          B|220     B|234     B|235     B|236     B|238     B|239     B|244
          B|245     B|246     B|247     B|248     B|249     B|250     B|253
          B|256     B|280     B|281     B|287     B|288     B|289     B|292
          B|293     B|294     B|295     B|318     B|319     B|327     B|328
          B|329     B|343     B|345     B|346
  
 Residues pruned out by function/max condition:
          A|149     A|187!    A|188     A|220!    A|259     A|319!    A|346!
         A|ENDA     B|149     B|187!    B|188     B|220!    B|259     B|319!
          B|346!   B|ENDA 
  
 Total number of residues for which LSSR restraints have been pruned out is      103 out of      698 participating
 Number of LSSR restraints pruned out is      7197 this is   20.7% of the total           34799
  
 -autoncs .Gelly cards suitable for adaptation for manual definition/pruning
           (note auto pruning is done in a slightly different way so exact results may differ slightly)
 -autoncs option .Gelly cards also written to file  auto_gelly_cards.txt
NOTE BUSTER_SET ncsautoXcld = Water    # card added - produced by -autoncs
NOTE BUSTER_SET ncsautoXcld = ncsautoXcld + { A|34 A|135 A|137 A|147 A|148 }    # card added - produced by -autoncs
NOTE BUSTER_SET ncsautoXcld = ncsautoXcld + { A|149 A|150 A|151 A|173 A|181 }    # card added - produced by -autoncs
NOTE BUSTER_SET ncsautoXcld = ncsautoXcld + { A|184 A|186 A|187 A|188 A|189 }    # card added - produced by -autoncs
NOTE BUSTER_SET ncsautoXcld = ncsautoXcld + { A|216 A|217 A|220 A|234 A|235 }    # card added - produced by -autoncs
NOTE BUSTER_SET ncsautoXcld = ncsautoXcld + { A|236 A|238 A|239 A|244 A|245 }    # card added - produced by -autoncs
NOTE BUSTER_SET ncsautoXcld = ncsautoXcld + { A|246 A|247 A|248 A|249 A|250 }    # card added - produced by -autoncs
NOTE BUSTER_SET ncsautoXcld = ncsautoXcld + { A|253 A|256 A|259 A|280 A|281 }    # card added - produced by -autoncs
NOTE BUSTER_SET ncsautoXcld = ncsautoXcld + { A|287 A|288 A|289 A|292 A|293 }    # card added - produced by -autoncs
NOTE BUSTER_SET ncsautoXcld = ncsautoXcld + { A|294 A|295 A|318 A|319 A|327 }    # card added - produced by -autoncs
NOTE BUSTER_SET ncsautoXcld = ncsautoXcld + { A|328 A|329 A|343 A|344 A|345 }    # card added - produced by -autoncs
NOTE BUSTER_SET ncsautoXcld = ncsautoXcld + { A|346 A|ENDA }    # card added - produced by -autoncs
NOTE BUSTER_SET ncsautoABset = Chain_A \ ncsautoXcld    # card added - produced by -autoncs
NOTE BUSTER_SIM_DEFINE ncsautoAB ncsautoABset B    # card added - produced by -autoncs
NOTE BUSTER_SIM_RESTRAIN_LSSR ncsautoAB 6.666    # card added - produced by -autoncs

 LSSR function contribution before pruning     46198.1
                             after pruning     10795.7
 Graph of which residues pruned from LSSR: ncs_pruned.mtv
 Graph of LSSR function outliers ncs_function_outliers.mtv
 Graph of LSSR gradient outliers ncs_gradient_outliers.mtv
 LSSR - analysis of distance difference distribution for NCS
         DeltaD less than 0.5 Angs:  proportion of population= 98.58% average DeltaD of subset     0.014 Angs
 Histogram of LSSR deltaD distribution ncs_histogram.mtv
 
 Interpreting card: NOTE BUSTER_TLS_SET tls1 { A|* }
 Interpreting card: NOTE BUSTER_TLS_SET tls2 { B|* }
 Interpreting card: NOTE BUSTER_TLS_SET tls1 { A|* }
 Interpreting card: NOTE BUSTER_TLS_T   tls1     -1.71079E-01 -7.47635E-02  2.43417E-01 -2.86465E-02  4.33199E-02  1.72722E-03
 Interpreting card: NOTE BUSTER_TLS_L   tls1      1.08059E+00  8.73620E-01  2.76036E-01  2.69469E-01 -4.48190E-01  2.11414E-01
 Interpreting card: NOTE BUSTER_TLS_S   tls1      2.37944E-01 -2.31254E-01  1.94103E-01 -1.31455E-01 -6.52398E-02 -1.27204E-01 -5.47966E-02 -8.78254E-02
 Interpreting card: NOTE BUSTER_TLS_O   tls1     -2.33533E+01 -8.36748E+01  1.13031E+01
 Interpreting card: NOTE BUSTER_TLS_SET tls2 { B|* }
 Interpreting card: NOTE BUSTER_TLS_T   tls2     -2.23653E-01 -1.74475E-01  3.03964E-01  1.65432E-02  5.01377E-02 -1.27065E-03
 Interpreting card: NOTE BUSTER_TLS_L   tls2      1.11201E+00  9.53119E-01  5.48649E-01  4.89954E-01 -5.70903E-01 -5.86900E-01
 Interpreting card: NOTE BUSTER_TLS_S   tls2     -3.19190E-02  1.25240E-01 -1.10983E-01  8.41507E-02  1.92000E-01  2.79219E-02 -6.18920E-02  9.91562E-02
 Interpreting card: NOTE BUSTER_TLS_O   tls2      2.20712E+01 -7.19054E+01  3.51200E+00
 Interpreting card: NOTE BUSTER_TLS_FIX tls1 ALL
 Interpreting card: NOTE BUSTER_TLS_FIX tls2 ALL


 TLS information. Total number of groups:     2


 Group   Natoms    FixRB   FixTLS   Group Tag    Group Spec
     1     2770      Y        Y     tls1    { A|* }
     2     2767      Y        Y     tls2    { B|* }
 
NOTE BUSTER_TLS_SET tls1 { A|* }
NOTE BUSTER_TLS_T   tls1     -1.71079E-01 -7.47635E-02  2.43417E-01 -2.86465E-02  4.33199E-02  1.72722E-03
NOTE BUSTER_TLS_L   tls1      1.08059E+00  8.73620E-01  2.76036E-01  2.69469E-01 -4.48190E-01  2.11414E-01
NOTE BUSTER_TLS_S   tls1      2.37944E-01 -2.31254E-01  1.94103E-01 -1.31455E-01 -6.52398E-02 -1.27204E-01 -5.47966E-02 -8.78254E-02
NOTE BUSTER_TLS_O   tls1     -2.33533E+01 -8.36748E+01  1.13031E+01
NOTE BUSTER_TLS_FIX tls1 ALL
 
NOTE BUSTER_TLS_SET tls2 { B|* }
NOTE BUSTER_TLS_T   tls2     -2.23653E-01 -1.74475E-01  3.03964E-01  1.65432E-02  5.01377E-02 -1.27065E-03
NOTE BUSTER_TLS_L   tls2      1.11201E+00  9.53119E-01  5.48649E-01  4.89954E-01 -5.70903E-01 -5.86900E-01
NOTE BUSTER_TLS_S   tls2     -3.19190E-02  1.25240E-01 -1.10983E-01  8.41507E-02  1.92000E-01  2.79219E-02 -6.18920E-02  9.91562E-02
NOTE BUSTER_TLS_O   tls2      2.20712E+01 -7.19054E+01  3.51200E+00
NOTE BUSTER_TLS_FIX tls2 ALL


 TLS: mmCIF group specification begin


#
loop_
_pdbx_refine_tls_group.id
_pdbx_refine_tls_group.refine_tls_id
_pdbx_refine_tls_group.beg_PDB_ins_code
_pdbx_refine_tls_group.beg_auth_asym_id
_pdbx_refine_tls_group.beg_auth_seq_id
_pdbx_refine_tls_group.end_PDB_ins_code
_pdbx_refine_tls_group.end_auth_asym_id
_pdbx_refine_tls_group.end_auth_seq_id
_pdbx_refine_tls_group.selection
_pdbx_refine_tls_group.selection_details
   1   1 . A   -1 . A  346 . '{ A|* }'
   2   1 . A  401 . A  401 . '{ A|* }'
   3   2 . B   -1 . B  346 . '{ B|* }'
   4   2 . B  401 . B  401 . '{ B|* }'
#


 TLS: mmCIF group specification end


 Interpreting card: NOTE BUSTER_ANISO_SET Empty
 Interpreting card: NOTE BUSTER_ANISO_WEIGHT SCA  16.0
 Interpreting card: NOTE BUSTER_ANISO_WEIGHT SPH   0.6    0.6
 Interpreting card: NOTE BUSTER_ANISO_WEIGHT SIM   7.8    4.8    2.6    0
 Interpreting card: NOTE BUSTER_ANISO_WEIGHT RIG  21.5   21.5    3.7    0


 After TLS and ANISO: Number of atoms with fixed/combined xyz =     0
 After TLS and ANISO: Number of atoms with fixed/combined B   =     0
 After TLS and ANISO: Number of atoms with fixed/combined OCC =  6503
 After TLS and ANISO: Number of atoms with fixed/combined BIJ =  6503
 After TLS and ANISO: Number of atoms in a TLS group          =  5537
                                     cf total number of atoms =  6503
 

 ############### Setup state vector

 No free_vect_ulim_xyz argument found: using default of  100.000
 No free_vect_llim_xyz argument found: using default of -100.000
 Number of parameters added for TLS description                 =       0
 Number of parameters to describe molecular conformation        =   26012

 ############### Setup free vector

 Number of free parameters to be adjusted in refinement   =   26012

 ############### Setting up own geometry


 No command line argument '-tnt_fn' so will use gelly to do geometry function calculations

 ============= Call TNT functions


 ============= Get TNT symmetry information

 
      Getting symmetry operators from TNT. 
          gelly will classify symmetry using pdb-like convention: 
               SYMOP   SYMMETRY
              NNNMMM   OPERATOR
                1555   X,Y,Z
                2555   -Y,X-Y,Z
                3555   -X+Y,-X,Z
                4555   -X,-Y,1/2+Z
                5555   Y,-X+Y,1/2+Z
                6555   X-Y,X,1/2+Z
          where NNN -> operator number and MMM -> translation vector
 

 ============= Own Gelly geometry/restraints setup


 ----------- Identify bond-types


    Setting up GEOMBONDREC_TYPE_HASH* to be able to map a given bond type (e.g.
    "single") to an internal integer (GBOND_TYPE=1) etc.

    Using default  GEOMBONDREC_TYPE_HASH_STR='SING* 1 DOUB* 2 TRIP* 3 AROM* 4 DELO* 4 METAL* 5':
    to override default use a NOTE BUSTER_GEOMBONDREC_TYPE_HASH_STR gelly card.

 ----------- Classify torsion into peptide omega and other


 ----------- Setup of non-bonded terms

 Ideal-distance contact term setup:
    Restraint function constants (normally these values are from NOTE BUSTER_IDEAL_CONTACT_* cards)
    Sigma for general contacts      0.200 Angs
    Sigma for 1-4 contacts          0.100 Angs (contacts across a torsion)
    Sigma for 1-5 contacts          0.150 Angs
    Sigma for 1-N contacts          0.200 Angs
    Sigma for HBOND pairs           0.100 Angs (contacts between HB_types A and D or B's)
    Contact distance increment for each atom in HBOND          -0.150 Angs 
    Contact distance increment for H-bond acceptor              0.100 Angs 
    Contact distance increment for 1-4 with an O atom          -0.050 Angs 
    Contact distance increment for 1-4 with an N atom          -0.180 Angs 
    Contact distance increment for 1-4 with an C atom          -0.370 Angs 
    Contact distance increment for 1-4 with an S atom          -0.150 Angs 
    Contact distance increment for 1-4 with an P atom          -0.150 Angs 
    Contact distance increment for 1-4 with any other atom     -0.150 Angs 
    All atoms matched for CCP4 atom type.
    Number of CCP4 atom types used is         24 (H-bound and not H-bound counted separately)

 Setting up short/ideal-distance contacts
   Using gelly special position handling. (For TNT instead rerun with argument '-special_tnt')

   No command line argument '-special_all' specified. We will auto-generate
   EXCLUDE cards only for atom-atom contacts across symmetry (i.e. on special
   position) when this involves a contact within a residue to itself.

   Have set up non-bonded exclusions - 1-3 contacts
   Atom A|27:CA         makes the most 1-3 contacts:  6

   Have set up non-bonded exclusions - 1-4 contacts
   Atom A|27:N          makes the most 1-4 contacts:  7

   Have set up non-bonded exclusions - 1-5 contacts
   Atom A|28:C.A        makes the most 1-5 contacts: 10

 ############################################################################################################
 ###################################################### setting up contact terms
 ############################################################################################################


 ============= Call Gelly versions of TNT functions

 WARNING: 1-4 contact without 1-(2,3)-4 data for A|401:O2(CIT)        and A|401:C3(CIT)        X-X
 WARNING: 1-4 contact without 1-(2,3)-4 data for A|401:C2(CIT)        and A|401:O6(CIT)        X-X
 WARNING: 1-4 contact without 1-(2,3)-4 data for A|401:O7(CIT)        and A|401:O5(CIT)        X-X
 WARNING: 1-4 contact without 1-(2,3)-4 data for A|401:C4(CIT)        and A|401:O6(CIT)        X-X
 WARNING: 1-4 contact without 1-(2,3)-4 data for B|401:C05(LIG)       and B|401:C08(LIG)       X-X
 WARNING: 1-4 contact without 1-(2,3)-4 data for B|401:C06(LIG)       and B|401:C09(LIG)       X-X

       TERM      weight      number      TNT_fn    Gelly_fn        diff
       BOND       2.000        5652    1839.276    1839.276      -0.000
       ANGL       2.000        7669    5642.255    5642.255       0.000
       IMPR       0.000           0       0.000       0.000       0.000
       TORS       2.000        1967    3307.061    3307.061      -0.000
       TRIG       2.000           0       0.000       0.000       0.000
       PLAN       5.000         963    2691.575    2691.575       0.000
       CONT       5.000           0     160.748       0.000     160.748
       BCOR      10.000        5630    6625.007   10607.381   -3982.375


 ============= Call Gelly geometry functions

   Switching torsion functional form to sinusoidal rather than split harmonic functional.
      if you want this not to happen then rerun with '-torsharm'
      Weight for sinusoidal torsion=     2.000
   BCOR function/gradient will be split between H and non-H atoms (as for any atom pair). Use '-bcorrel_all_in_hydrogens' to change this.

 ----------- Fix B-correlation restraints

 
 Checking for bonds that do not have a bcorrel restraint defined
 We will wipe all existing BCORrels if OCC=0.0 atom(s) is/are involved. This can be' switched off via the '-bcorrel_nowipe_zeroocc' flag.
 We will wipe all existing BCORrels if only non-hydrogen atom(s) with fixed B(iso) are involved. This can be switched off via the '-bcorrel_nowipe_fixb_nonhydrogens' flag.
 We will keep all existing BCORrels if hydrogen atom(s) with fixed B(iso) is/are involved. This can be switched off via the '-bcorrel_wipe_fixb_hydrogens' flag.
 No -bcorrel_missing_sigma found. So will set sigma to     2.5 Ang**2 for any bond with no existing BCORrel
 No -bcorrel_missing_sigma_hydrogens found. So will set sigma to     1.0 Ang**2 for any bond with no existing BCORrel involving hydrogens

 No active BCORels found involving OCC=0.0 atom(s)


 No active BCORels found involving fixed-B (only) non-hydrogen atom(s)

    add a default BCOR for bond A|401:C1(CIT) A|401:C2(CIT)
    add a default BCOR for bond A|401:C1(CIT) A|401:O1(CIT)
    add a default BCOR for bond A|401:C1(CIT) A|401:O2(CIT)
    add a default BCOR for bond A|401:C2(CIT) A|401:C3(CIT)
    add a default BCOR for bond A|401:C3(CIT) A|401:C4(CIT)
    add a default BCOR for bond A|401:C3(CIT) A|401:C6(CIT)
    add a default BCOR for bond A|401:C3(CIT) A|401:O7(CIT)
    add a default BCOR for bond A|401:C4(CIT) A|401:C5(CIT)
    add a default BCOR for bond A|401:C5(CIT) A|401:O3(CIT)
    add a default BCOR for bond A|401:C5(CIT) A|401:O4(CIT)
    add a default BCOR for bond A|401:C6(CIT) A|401:O5(CIT)
    add a default BCOR for bond A|401:C6(CIT) A|401:O6(CIT)
    add a default BCOR for bond B|401:C01(LIG) B|401:N02(LIG)
    add a default BCOR for bond B|401:N02(LIG) B|401:C03(LIG)
    add a default BCOR for bond B|401:C03(LIG) B|401:O04(LIG)
    add a default BCOR for bond B|401:C03(LIG) B|401:C05(LIG)
    add a default BCOR for bond B|401:C05(LIG) B|401:C06(LIG)
    add a default BCOR for bond B|401:C05(LIG) B|401:O10(LIG)
    add a default BCOR for bond B|401:C06(LIG) B|401:N07(LIG)
    add a default BCOR for bond B|401:N07(LIG) B|401:C08(LIG)
    add a default BCOR for bond B|401:C08(LIG) B|401:C09(LIG)
    add a default BCOR for bond B|401:C09(LIG) B|401:O10(LIG)

 Have added a total of        22 BCORrels for bonds which do not already have a BCORrel


 No bonds (for potential BCORrel addition) were skipped due to both atoms having constant/fixed B(iso)


 ----------- Deactivate hydrogen-free planes if hydrogen atoms present

 A total of       0 planes have been deactivated because they are duplicated hydrogen-free and with-hydrogen forms.

 ----------- Handle peptide omega angle(s)

      Torsion angles split into peptide omega and other
         Number of peptide omega angles is       698
         Number of other torsion is             1269
      Peptide omega angles are monitored rather than directly restrained (PLANE used instead).

 ----------- Fix cis-PRO

 Now apply the fix to get correct restraints for cis PRO residues
   Have got      11 bonds records and      26 angles
   from GEOMETRY cards for the special PROC residue
   Weight for ideal-distance contact term=     4.000

 ----------- Prepare SSBOND/LINK for output PDB file


 ----------- Prepare for QM


 ----------- Prepare symmetry in utility distance restraints


 ############### Setting up Rhofit contacts (if needed)

 No ligand-(fixed protein) contact term will be used

 ############### Working out initial function and gradient


   MaxLik Scaling Cycle 0002
 
 Initial functional value = 4.171391E+05 rms gradient=      59.8967
 X-ray contribution to function value = 3.712114E+05
 Geometry contribution (inc ncs)      =      45927.6
 Geometry function summary
     term                           number     weight       rms        GooF      function     fn/numb
     ================================================================================================
     BOND bond lengths (angs)       5652       2.000      0.0081       0.403      1839.28       0.325
     ANGL bond angles (degs)        7669       2.000        1.02       0.607      5642.25       0.736
     TORS split-harmonic tors       1269       0.000       18.14       1.141         0.00       0.000
     SINTOR sinusoidal tors         1269       2.000       18.14                  2488.63       1.961
     PLAN general plane (angs)       963       5.000      0.0150       0.748      2691.58       2.795
     BCORrelations (angs^2)         5652      10.000       1.214       0.433     10616.37       1.878
     CONT (bad contacts)               0       5.000                                 0.00
     IDEAL(ideal-dist contact)      5474       4.000                             11794.00
     CHIRAL (gelly semiharmon)       740       5.000                                59.86
     SIM similarity restraint for NCS and/or TARGET                              10795.65
     OCCSUM restraint on sum of occupanies                                           0.00       as none defined
     DISTAN utility restraints interatomic distance                                  0.00       as none defined
     UTILANGLE utility angle restraints                                              0.00       as none defined
     UTILTOR utility restraints on torsion angles                                    0.00       as none defined
     Peptide omega torsion angles                           3.03 degs - monitor only

 Weighted rms      0.0081         1.018  (temporary rms bond and angle for BUSTER to pick up!)
 Weighted intra-residual rms(bond) for StdProtein (excluding H)      0.0079
 Weighted intra-residual rms(bond) for Buffer     (excluding H)      0.0033

 ############### Running initial check


 Initial Geometry Sanity Check

 Check for duplicate bonds: no duplicates found
 
 Check for duplicate or missing bond angles: all are OK
 Check for bond between an atom and itself not involving symmetry:  all are OK
   There are no bad contacts where Delta/sigma is greater than     6.5
   Worst contact dist=   3.015 std=   3.836 sigma=   0.217 abs(Delta/sigma)=    3.782 ats A|1:CB (SER) B|1:CB (SER)
   It is likely that the geometry libraries, sequence info and links match the input molecule
 GEOMETRY CHECK OVERALL:  PASS


 ############### Creating geometry/screen report


 ############### Option "tao" - run Tao optimizer

 TAO: Toolkit for Advanced Optimization. Reference: 
     Munson, T., Sarich, J., Wild, S., Benson, S., McInnes, L.C. (2018)
     "TAO User Manual (Revision 3.9)", Mathematics and Computer Science
     Division, Argonne National Laboratory
     ANL/MCS-TM-322, http://www.mcs.anl.gov/petsc/
 
 Have found -keeppdb flag so will write out a .pdb file every 1000000 its during opt
 Will apply TAO optimization 
 Maximum number of iterations      100
 Convergence test on Grms       4.0000 (-glim)
 Limit on rms displacement to original position (rmsD2init): NONE test inactive (-dlim)

    analysing argument no 0 : -autoncs with j=0
    analysing argument no 1 : -hydrogens_input_occ2zero with j=0
    analysing argument no 2 : -hydrogens_output_occ2heavy with j=0
    analysing argument no 3 : -screen with j=0
    analysing argument no 4 : 100 with j=0
    analysing argument no 5 : -screen_sigma with j=0
    analysing argument no 6 : 3.0 with j=0
    analysing argument no 7 : -keeppdb with j=0
    analysing argument no 8 : 1000000 with j=0
    analysing argument no 9 : -taomonitor_verbose with j=0
    found argument starting with "-tao" : -taomonitor_verbose
    analysing argument no 10 : 2 with j=1
    found value after -tao/-options_/-viewer_ argument : "2"
 PETSC/TAO: setting option "-taomonitor_verbose" = "2"


   MaxLik Scaling Cycle 0003
   Iters     Tot_Funct    Xray_Funct    Geom_Funct          Grms     rmsD2init     maxD2init             R         Rfree           LLG       LLGfree     rms(bond)    rms(angle)       Opttag_H
   Iters             K   B_IMPF_FRAG        K_MISS   B_TEMP_MISS   K_IMPF_MISS   B_IMPF_MISS        K_SOLV   B_TEMP_SOLV   K_IMPF_SOLV   B_IMPF_SOLV        B_TEMP     B_TEMP_11     B_TEMP_12     B_TEMP_22     B_TEMP_13     B_TEMP_23     B_TEMP_33      K_WILSON      B_WILSON      FGCYCLES       Scatag_H
   Iters          BOND         ANGLE      IMPROPER       TORSION    SINTORSION      TRIGONAL        PLANAR  BCORRELATION       CONTACT     IDEALDIST        CHIRAL    SIMILARITY       OCCSSUM  UTILDISTANCE     UTILANGLE   UTILTORSION        RHOFIT        ADP-01        ADP-02        ADP-03        ADP-04        ADP-05        ADP-06        ADP-07        ADP-08        ADP-09        ADP-10      RHOFITPC            QM       Funtag_H
       0  4.171391E+05  3.712114E+05       45927.6       59.8967  0.000000E+00  0.000000E+00      0.191053      0.234691  0.000000E+00  0.000000E+00    0.00809419       1.01755       Opttag_D
       0       78.4234       1.56518       1.00000  0.000000E+00       1.00000  0.000000E+00      0.885379       84.1907       1.00000       32.1317  0.000000E+00      0.720420  0.000000E+00      0.720420  0.000000E+00  0.000000E+00      -1.44084       79.3031       1.08731        2       Scatag_D
       0       1839.28       5642.25  0.000000E+00  0.000000E+00       2488.63  0.000000E+00       2691.58       10616.4  0.000000E+00       11794.0       59.8556       10795.7 -1.000000E+10 -1.000000E+10 -1.000000E+10 -1.000000E+10 -1.000000E+10  0.000000E+00  0.000000E+00  0.000000E+00  0.000000E+00  0.000000E+00  0.000000E+00  0.000000E+00  0.000000E+00  0.000000E+00  0.000000E+00  0.000000E+00  0.000000E+00       Funtag_D
   Geometry Restraint Deviations SCREEN Information

 

 An atom has moved more than     1.50 A from the initial/last
 position - if this was an accepted trial point, probably need to update the
 non-bonded contact list.


   MaxLik Scaling Cycle 0004

   MaxLik Scaling Cycle 0005

   MaxLik Scaling Cycle 0006

   Wilson plot

   Statistics in resolution bins after refinement cycle 0001
```


|  |  |
| --- | --- |
|  | - Rxpct factors plot- Rxpct factors table- <Log-Lik Gain>- Ln Sigma A- Correlation Coefficients |


```
   Iters     Tot_Funct    Xray_Funct    Geom_Funct          Grms     rmsD2init     maxD2init             R         Rfree           LLG       LLGfree     rms(bond)    rms(angle)
   Iters             K   B_IMPF_FRAG        K_MISS   B_TEMP_MISS   K_IMPF_MISS   B_IMPF_MISS        K_SOLV   B_TEMP_SOLV   K_IMPF_SOLV   B_IMPF_SOLV        B_TEMP     B_TEMP_11     B_TEMP_12     B_TEMP_22     B_TEMP_13     B_TEMP_23     B_TEMP_33      K_WILSON      B_WILSON      FGCYCLES
   Iters          BOND         ANGLE      IMPROPER       TORSION    SINTORSION      TRIGONAL        PLANAR  BCORRELATION       CONTACT     IDEALDIST        CHIRAL    SIMILARITY       OCCSSUM  UTILDISTANCE     UTILANGLE   UTILTORSION        RHOFIT        ADP-01        ADP-02        ADP-03        ADP-04        ADP-05        ADP-06        ADP-07        ADP-08        ADP-09        ADP-10      RHOFITPC            QM
       1  4.163624E+05  3.710933E+05       45269.1       50.0861  4.809260E-04    0.00477996      0.191025      0.234681  2.871183E-04  2.112358E-04    0.00805174       1.01785       Opttag_D
       1       78.3832       1.55803       1.00000  0.000000E+00       1.00000  0.000000E+00      0.885435       83.9863       1.00000       32.1298  0.000000E+00      0.731438  0.000000E+00      0.731438  0.000000E+00  0.000000E+00      -1.46288       79.3031       1.08731        3       Scatag_D
       1       1820.04       5645.58  0.000000E+00  0.000000E+00       2488.36  0.000000E+00       2689.94       9998.51  0.000000E+00       11794.1       59.6686       10772.9 -1.000000E+10 -1.000000E+10 -1.000000E+10 -1.000000E+10 -1.000000E+10  0.000000E+00  0.000000E+00  0.000000E+00  0.000000E+00  0.000000E+00  0.000000E+00  0.000000E+00  0.000000E+00  0.000000E+00  0.000000E+00  0.000000E+00  0.000000E+00       Funtag_D
   Geometry Restraint Deviations SCREEN Information

 

   MaxLik Scaling Cycle 0007

   Wilson plot

   Statistics in resolution bins after refinement cycle 0002
```


|  |  |
| --- | --- |
|  | - Rxpct factors plot- Rxpct factors table- <Log-Lik Gain>- Ln Sigma A- Correlation Coefficients |


```
   Iters     Tot_Funct    Xray_Funct    Geom_Funct          Grms     rmsD2init     maxD2init             R         Rfree           LLG       LLGfree     rms(bond)    rms(angle)
   Iters             K   B_IMPF_FRAG        K_MISS   B_TEMP_MISS   K_IMPF_MISS   B_IMPF_MISS        K_SOLV   B_TEMP_SOLV   K_IMPF_SOLV   B_IMPF_SOLV        B_TEMP     B_TEMP_11     B_TEMP_12     B_TEMP_22     B_TEMP_13     B_TEMP_23     B_TEMP_33      K_WILSON      B_WILSON      FGCYCLES
   Iters          BOND         ANGLE      IMPROPER       TORSION    SINTORSION      TRIGONAL        PLANAR  BCORRELATION       CONTACT     IDEALDIST        CHIRAL    SIMILARITY       OCCSSUM  UTILDISTANCE     UTILANGLE   UTILTORSION        RHOFIT        ADP-01        ADP-02        ADP-03        ADP-04        ADP-05        ADP-06        ADP-07        ADP-08        ADP-09        ADP-10      RHOFITPC            QM
       2  4.158423E+05  3.709824E+05       44859.9       38.3500  9.707993E-04     0.0108464      0.191004      0.234727  5.561594E-04  2.511680E-04    0.00803228       1.01821       Opttag_D
       2       78.3363       1.55070       1.00000  0.000000E+00       1.00000  0.000000E+00      0.885446       83.7859       1.00000       32.1221  0.000000E+00      0.743063  0.000000E+00      0.743063  0.000000E+00  0.000000E+00      -1.48613       79.3031       1.08731        1       Scatag_D
       2       1811.25       5649.63  0.000000E+00  0.000000E+00       2488.25  0.000000E+00       2688.03       9617.56  0.000000E+00       11794.8       59.4968       10750.8 -1.000000E+10 -1.000000E+10 -1.000000E+10 -1.000000E+10 -1.000000E+10  0.000000E+00  0.000000E+00  0.000000E+00  0.000000E+00  0.000000E+00  0.000000E+00  0.000000E+00  0.000000E+00  0.000000E+00  0.000000E+00  0.000000E+00  0.000000E+00       Funtag_D
   Geometry Restraint Deviations SCREEN Information

 
       3  4.154012E+05  3.708315E+05       44569.6       38.2267    0.00173511     0.0209860      0.190979      0.234789  9.222247E-04  2.216529E-04    0.00802317       1.01950       Opttag_D
       3       78.2634       1.54011       1.00000  0.000000E+00       1.00000  0.000000E+00      0.885440       83.4924       1.00000       32.1100  0.000000E+00      0.759833  0.000000E+00      0.759833  0.000000E+00  0.000000E+00      -1.51967       79.3031       1.08731        1       Scatag_D
       3       1807.14       5663.96  0.000000E+00  0.000000E+00       2487.92  0.000000E+00       2685.80       9345.84  0.000000E+00       11797.4       59.4066       10722.2 -1.000000E+10 -1.000000E+10 -1.000000E+10 -1.000000E+10 -1.000000E+10  0.000000E+00  0.000000E+00  0.000000E+00  0.000000E+00  0.000000E+00  0.000000E+00  0.000000E+00  0.000000E+00  0.000000E+00  0.000000E+00  0.000000E+00  0.000000E+00       Funtag_D
       4  4.151053E+05  3.706599E+05       44445.5       56.0012    0.00278927     0.0365281      0.190966      0.234851    0.00133885  1.773389E-04    0.00805089       1.02611       Opttag_D
       4       78.1611       1.52654       1.00000  0.000000E+00       1.00000  0.000000E+00      0.885403       83.1015       1.00000       32.0971  0.000000E+00      0.782611  0.000000E+00      0.782611  0.000000E+00  0.000000E+00      -1.56522       79.3031       1.08731        1       Scatag_D
       4       1819.65       5737.64  0.000000E+00  0.000000E+00       2487.40  0.000000E+00       2683.99       9162.53  0.000000E+00       11800.1       59.3990       10694.7 -1.000000E+10 -1.000000E+10 -1.000000E+10 -1.000000E+10 -1.000000E+10  0.000000E+00  0.000000E+00  0.000000E+00  0.000000E+00  0.000000E+00  0.000000E+00  0.000000E+00  0.000000E+00  0.000000E+00  0.000000E+00  0.000000E+00  0.000000E+00       Funtag_D
       5  4.147951E+05  3.705923E+05       44202.8       34.1489    0.00315127     0.0432842      0.190963      0.234870    0.00150275  1.405483E-04    0.00802456       1.01953       Opttag_D
       5       78.1194       1.52121       1.00000  0.000000E+00       1.00000  0.000000E+00      0.885373       82.9554       1.00000       32.0929  0.000000E+00      0.791233  0.000000E+00      0.791233  0.000000E+00  0.000000E+00      -1.58247       79.3031       1.08731        1       Scatag_D
       5       1807.77       5664.27  0.000000E+00  0.000000E+00       2487.22  0.000000E+00       2683.67       9015.91  0.000000E+00       11800.5       59.4397       10684.0 -1.000000E+10 -1.000000E+10 -1.000000E+10 -1.000000E+10 -1.000000E+10  0.000000E+00  0.000000E+00  0.000000E+00  0.000000E+00  0.000000E+00  0.000000E+00  0.000000E+00  0.000000E+00  0.000000E+00  0.000000E+00  0.000000E+00  0.000000E+00       Funtag_D
       6  4.144651E+05  3.704882E+05       43976.8       35.2743    0.00378279     0.0550574      0.190964      0.234889    0.00175526  7.415989E-05    0.00802178       1.02051       Opttag_D
       6       78.0496       1.51253       1.00000  0.000000E+00       1.00000  0.000000E+00      0.885313       82.7182       1.00000       32.0882  0.000000E+00      0.805369  0.000000E+00      0.805369  0.000000E+00  0.000000E+00      -1.61074       79.3031       1.08731        1       Scatag_D
       6       1806.52       5675.12  0.000000E+00  0.000000E+00       2486.78  0.000000E+00       2683.76       8796.55  0.000000E+00       11800.3       59.5917       10668.2 -1.000000E+10 -1.000000E+10 -1.000000E+10 -1.000000E+10 -1.000000E+10  0.000000E+00  0.000000E+00  0.000000E+00  0.000000E+00  0.000000E+00  0.000000E+00  0.000000E+00  0.000000E+00  0.000000E+00  0.000000E+00  0.000000E+00  0.000000E+00       Funtag_D
       7  4.141438E+05  3.703789E+05       43764.9       42.1095    0.00472225     0.0722870      0.190987      0.234934    0.00202063 -5.415242E-05    0.00802848       1.02325       Opttag_D
       7       77.9521       1.50118       1.00000  0.000000E+00       1.00000  0.000000E+00      0.885227       82.3804       1.00000       32.0847  0.000000E+00      0.825497  0.000000E+00      0.825497  0.000000E+00  0.000000E+00      -1.65099       79.3031       1.08731        1       Scatag_D
       7       1809.54       5705.62  0.000000E+00  0.000000E+00       2486.12  0.000000E+00       2685.36       8568.89  0.000000E+00       11799.1       59.8635       10650.4 -1.000000E+10 -1.000000E+10 -1.000000E+10 -1.000000E+10 -1.000000E+10  0.000000E+00  0.000000E+00  0.000000E+00  0.000000E+00  0.000000E+00  0.000000E+00  0.000000E+00  0.000000E+00  0.000000E+00  0.000000E+00  0.000000E+00  0.000000E+00       Funtag_D
       8  4.139312E+05  3.702977E+05       43633.5       63.8577    0.00647638      0.103459      0.191085      0.235083    0.00221772 -3.858464E-04    0.00808930       1.03147       Opttag_D
       8       77.7867       1.48432       1.00000  0.000000E+00       1.00000  0.000000E+00      0.885074       81.7974       1.00000       32.0869  0.000000E+00      0.860053  0.000000E+00      0.860053  0.000000E+00  0.000000E+00      -1.72011       79.3031       1.08731        1       Scatag_D
       8       1837.05       5797.69  0.000000E+00  0.000000E+00       2485.08  0.000000E+00       2691.40       8332.14  0.000000E+00       11798.7       60.3964       10631.1 -1.000000E+10 -1.000000E+10 -1.000000E+10 -1.000000E+10 -1.000000E+10  0.000000E+00  0.000000E+00  0.000000E+00  0.000000E+00  0.000000E+00  0.000000E+00  0.000000E+00  0.000000E+00  0.000000E+00  0.000000E+00  0.000000E+00  0.000000E+00       Funtag_D
       9  4.136426E+05  3.702841E+05       43358.5       27.0589    0.00652105      0.104784      0.191087      0.235089    0.00225048 -3.924604E-04    0.00803898       1.01962       Opttag_D
       9       77.7782       1.48301       1.00000  0.000000E+00       1.00000  0.000000E+00      0.885068       81.7599       1.00000       32.0900  0.000000E+00      0.861395  0.000000E+00      0.861395  0.000000E+00  0.000000E+00      -1.72279       79.3031       1.08731        1       Scatag_D
       9       1814.27       5665.23  0.000000E+00  0.000000E+00       2484.83  0.000000E+00       2692.17       8219.83  0.000000E+00       11795.6       60.3290       10626.2 -1.000000E+10 -1.000000E+10 -1.000000E+10 -1.000000E+10 -1.000000E+10  0.000000E+00  0.000000E+00  0.000000E+00  0.000000E+00  0.000000E+00  0.000000E+00  0.000000E+00  0.000000E+00  0.000000E+00  0.000000E+00  0.000000E+00  0.000000E+00       Funtag_D
      10  4.134933E+05  3.702659E+05       43227.5       24.5649    0.00666524      0.107462      0.191093      0.235113    0.00229483 -4.152788E-04    0.00803108       1.01974       Opttag_D
      10       77.7619       1.48077       1.00000  0.000000E+00       1.00000  0.000000E+00      0.885055       81.6906       1.00000       32.0948  0.000000E+00      0.864140  0.000000E+00      0.864140  0.000000E+00  0.000000E+00      -1.72828       79.3031       1.08731        1       Scatag_D
      10       1810.71       5666.64  0.000000E+00  0.000000E+00       2484.57  0.000000E+00       2693.67       8099.16  0.000000E+00       11792.4       60.2520       10620.0 -1.000000E+10 -1.000000E+10 -1.000000E+10 -1.000000E+10 -1.000000E+10  0.000000E+00  0.000000E+00  0.000000E+00  0.000000E+00  0.000000E+00  0.000000E+00  0.000000E+00  0.000000E+00  0.000000E+00  0.000000E+00  0.000000E+00  0.000000E+00       Funtag_D
      11  4.133148E+05  3.702431E+05       43071.7       27.5697    0.00723915      0.116783      0.191132      0.235186    0.00234998 -5.188712E-04    0.00803240       1.01992       Opttag_D
      11       77.7096       1.47494       1.00000  0.000000E+00       1.00000  0.000000E+00      0.885000       81.4855       1.00000       32.1046  0.000000E+00      0.873721  0.000000E+00      0.873721  0.000000E+00  0.000000E+00      -1.74744       79.3031       1.08731        1       Scatag_D
      11       1811.30       5668.57  0.000000E+00  0.000000E+00       2483.99  0.000000E+00       2697.90       7952.32  0.000000E+00       11788.0       60.2322       10609.4 -1.000000E+10 -1.000000E+10 -1.000000E+10 -1.000000E+10 -1.000000E+10  0.000000E+00  0.000000E+00  0.000000E+00  0.000000E+00  0.000000E+00  0.000000E+00  0.000000E+00  0.000000E+00  0.000000E+00  0.000000E+00  0.000000E+00  0.000000E+00       Funtag_D
      12  4.132334E+05  3.702305E+05       43002.9       63.9860    0.00849608      0.133861      0.191229      0.235332    0.00238077 -6.825686E-04    0.00807996       1.03246       Opttag_D
      12       77.6121       1.46462       1.00000  0.000000E+00       1.00000  0.000000E+00      0.884868       81.0960       1.00000       32.1277  0.000000E+00      0.891186  0.000000E+00      0.891186  0.000000E+00  0.000000E+00      -1.78237       79.3031       1.08731        1       Scatag_D
      12       1832.82       5808.87  0.000000E+00  0.000000E+00       2482.77  0.000000E+00       2707.83       7735.48  0.000000E+00       11780.9       60.1914       10594.1 -1.000000E+10 -1.000000E+10 -1.000000E+10 -1.000000E+10 -1.000000E+10  0.000000E+00  0.000000E+00  0.000000E+00  0.000000E+00  0.000000E+00  0.000000E+00  0.000000E+00  0.000000E+00  0.000000E+00  0.000000E+00  0.000000E+00  0.000000E+00       Funtag_D
      13  4.130098E+05  3.702305E+05       42779.3       23.6016    0.00871725      0.136496      0.191247      0.235363    0.00238070 -7.120837E-04    0.00804596       1.01834       Opttag_D
      13       77.5961       1.46296       1.00000  0.000000E+00       1.00000  0.000000E+00      0.884837       81.0313       1.00000       32.1311  0.000000E+00      0.893879  0.000000E+00      0.893879  0.000000E+00  0.000000E+00      -1.78776       79.3031       1.08731        1       Scatag_D
      13       1817.42       5651.04  0.000000E+00  0.000000E+00       2482.67  0.000000E+00       2709.42       7687.35  0.000000E+00       11779.5       60.1880       10591.7 -1.000000E+10 -1.000000E+10 -1.000000E+10 -1.000000E+10 -1.000000E+10  0.000000E+00  0.000000E+00  0.000000E+00  0.000000E+00  0.000000E+00  0.000000E+00  0.000000E+00  0.000000E+00  0.000000E+00  0.000000E+00  0.000000E+00  0.000000E+00       Funtag_D
      14  4.129207E+05  3.702221E+05       42698.6       16.8272    0.00894243      0.138343      0.191260      0.235380    0.00240103 -7.034028E-04    0.00804439       1.01754       Opttag_D
      14       77.5818       1.46118       1.00000  0.000000E+00       1.00000  0.000000E+00      0.884802       80.9695       1.00000       32.1343  0.000000E+00      0.896027  0.000000E+00      0.896027  0.000000E+00  0.000000E+00      -1.79205       79.3031       1.08731        1       Scatag_D
      14       1816.71       5642.14  0.000000E+00  0.000000E+00       2482.51  0.000000E+00       2710.67       7619.17  0.000000E+00       11778.2       60.1836       10589.0 -1.000000E+10 -1.000000E+10 -1.000000E+10 -1.000000E+10 -1.000000E+10  0.000000E+00  0.000000E+00  0.000000E+00  0.000000E+00  0.000000E+00  0.000000E+00  0.000000E+00  0.000000E+00  0.000000E+00  0.000000E+00  0.000000E+00  0.000000E+00       Funtag_D
      15  4.128179E+05  3.702132E+05       42604.7       20.5978    0.00950497      0.142870      0.191295      0.235427    0.00242257 -7.047256E-04    0.00805153       1.01800       Opttag_D
      15       77.5490       1.45725       1.00000  0.000000E+00       1.00000  0.000000E+00      0.884725       80.8212       1.00000       32.1438  0.000000E+00      0.901056  0.000000E+00      0.901056  0.000000E+00  0.000000E+00      -1.80211       79.3031       1.08731        1       Scatag_D
      15       1819.94       5647.29  0.000000E+00  0.000000E+00       2482.09  0.000000E+00       2714.17       7521.68  0.000000E+00       11775.5       60.2270       10583.8 -1.000000E+10 -1.000000E+10 -1.000000E+10 -1.000000E+10 -1.000000E+10  0.000000E+00  0.000000E+00  0.000000E+00  0.000000E+00  0.000000E+00  0.000000E+00  0.000000E+00  0.000000E+00  0.000000E+00  0.000000E+00  0.000000E+00  0.000000E+00       Funtag_D
      16  4.126908E+05  3.701969E+05       42494.0       28.1045     0.0103371      0.147121      0.191339      0.235484    0.00246226 -6.751278E-04    0.00806063       1.01940       Opttag_D
      16       77.5117       1.45198       1.00000  0.000000E+00       1.00000  0.000000E+00      0.884618       80.6147       1.00000       32.1573  0.000000E+00      0.905907  0.000000E+00      0.905907  0.000000E+00  0.000000E+00      -1.81181       79.3031       1.08731        1       Scatag_D
      16       1824.06       5662.78  0.000000E+00  0.000000E+00       2481.64  0.000000E+00       2717.82       7398.31  0.000000E+00       11772.1       60.3784       10576.8 -1.000000E+10 -1.000000E+10 -1.000000E+10 -1.000000E+10 -1.000000E+10  0.000000E+00  0.000000E+00  0.000000E+00  0.000000E+00  0.000000E+00  0.000000E+00  0.000000E+00  0.000000E+00  0.000000E+00  0.000000E+00  0.000000E+00  0.000000E+00       Funtag_D
      17  4.125782E+05  3.702047E+05       42373.5       24.6917     0.0115485      0.152209      0.191409      0.235569    0.00244333 -6.979462E-04    0.00807550       1.01816       Opttag_D
      17       77.4691       1.44603       1.00000  0.000000E+00       1.00000  0.000000E+00      0.884474       80.3444       1.00000       32.1800  0.000000E+00      0.910950  0.000000E+00      0.910950  0.000000E+00  0.000000E+00      -1.82190       79.3031       1.08731        1       Scatag_D
      17       1830.79       5649.10  0.000000E+00  0.000000E+00       2480.96  0.000000E+00       2723.81       7288.07  0.000000E+00       11768.9       60.6150       10571.3 -1.000000E+10 -1.000000E+10 -1.000000E+10 -1.000000E+10 -1.000000E+10  0.000000E+00  0.000000E+00  0.000000E+00  0.000000E+00  0.000000E+00  0.000000E+00  0.000000E+00  0.000000E+00  0.000000E+00  0.000000E+00  0.000000E+00  0.000000E+00       Funtag_D
      18  4.124939E+05  3.701917E+05       42302.2       17.4894     0.0119221      0.151798      0.191415      0.235585    0.00247490 -6.938124E-04    0.00806893       1.01752       Opttag_D
      18       77.4664       1.44441       1.00000  0.000000E+00       1.00000  0.000000E+00      0.884431       80.2711       1.00000       32.1870  0.000000E+00      0.909945  0.000000E+00      0.909945  0.000000E+00  0.000000E+00      -1.81989       79.3031       1.08731        1       Scatag_D
      18       1827.81       5641.95  0.000000E+00  0.000000E+00       2481.03  0.000000E+00       2723.67       7230.51  0.000000E+00       11768.1       60.6961       10568.5 -1.000000E+10 -1.000000E+10 -1.000000E+10 -1.000000E+10 -1.000000E+10  0.000000E+00  0.000000E+00  0.000000E+00  0.000000E+00  0.000000E+00  0.000000E+00  0.000000E+00  0.000000E+00  0.000000E+00  0.000000E+00  0.000000E+00  0.000000E+00       Funtag_D
      19  4.124192E+05  3.701641E+05       42255.1       16.8345     0.0121812      0.149760      0.191405      0.235589    0.00254177 -6.869504E-04    0.00806225       1.01779       Opttag_D
      19       77.4746       1.44338       1.00000  0.000000E+00       1.00000  0.000000E+00      0.884403       80.2292       1.00000       32.1914  0.000000E+00      0.906798  0.000000E+00      0.906798  0.000000E+00  0.000000E+00      -1.81360       79.3031       1.08731        1       Scatag_D
      19       1824.79       5644.92  0.000000E+00  0.000000E+00       2481.12  0.000000E+00       2721.61       7188.99  0.000000E+00       11767.5       60.7666       10565.4 -1.000000E+10 -1.000000E+10 -1.000000E+10 -1.000000E+10 -1.000000E+10  0.000000E+00  0.000000E+00  0.000000E+00  0.000000E+00  0.000000E+00  0.000000E+00  0.000000E+00  0.000000E+00  0.000000E+00  0.000000E+00  0.000000E+00  0.000000E+00       Funtag_D
      20  4.123466E+05  3.701286E+05       42218.0       19.9749     0.0128597      0.147528      0.191398      0.235625    0.00262792 -7.376304E-04    0.00806442       1.01816       Opttag_D
      20       77.4860       1.44131       1.00000  0.000000E+00       1.00000  0.000000E+00      0.884328       80.1268       1.00000       32.2041  0.000000E+00      0.901907  0.000000E+00      0.901907  0.000000E+00  0.000000E+00      -1.80381       79.3031       1.08731        1       Scatag_D
      20       1825.77       5649.00  0.000000E+00  0.000000E+00       2481.34  0.000000E+00       2719.98       7151.75  0.000000E+00       11767.0       60.8889       10562.2 -1.000000E+10 -1.000000E+10 -1.000000E+10 -1.000000E+10 -1.000000E+10  0.000000E+00  0.000000E+00  0.000000E+00  0.000000E+00  0.000000E+00  0.000000E+00  0.000000E+00  0.000000E+00  0.000000E+00  0.000000E+00  0.000000E+00  0.000000E+00       Funtag_D
      21  4.122719E+05  3.700869E+05       42185.0       16.9886     0.0134557      0.144983      0.191379      0.235648    0.00272925 -7.922789E-04    0.00805949       1.01810       Opttag_D
      21       77.5057       1.43995       1.00000  0.000000E+00       1.00000  0.000000E+00      0.884262       80.0581       1.00000       32.2163  0.000000E+00      0.895958  0.000000E+00      0.895958  0.000000E+00  0.000000E+00      -1.79192       79.3031       1.08731        1       Scatag_D
      21       1823.54       5648.43  0.000000E+00  0.000000E+00       2481.60  0.000000E+00       2718.06       7126.07  0.000000E+00       11766.1       60.9385       10560.3 -1.000000E+10 -1.000000E+10 -1.000000E+10 -1.000000E+10 -1.000000E+10  0.000000E+00  0.000000E+00  0.000000E+00  0.000000E+00  0.000000E+00  0.000000E+00  0.000000E+00  0.000000E+00  0.000000E+00  0.000000E+00  0.000000E+00  0.000000E+00       Funtag_D
      22  4.122021E+05  3.700488E+05       42153.3       14.4397     0.0139582      0.143386      0.191361      0.235661    0.00282161 -8.437857E-04    0.00805844       1.01815       Opttag_D
      22       77.5249       1.43897       1.00000  0.000000E+00       1.00000  0.000000E+00      0.884205       80.0095       1.00000       32.2277  0.000000E+00      0.890919  0.000000E+00      0.890919  0.000000E+00  0.000000E+00      -1.78184       79.3031       1.08731        1       Scatag_D
      22       1823.06       5648.92  0.000000E+00  0.000000E+00       2481.85  0.000000E+00       2717.02       7095.71  0.000000E+00       11766.2       60.9324       10559.7 -1.000000E+10 -1.000000E+10 -1.000000E+10 -1.000000E+10 -1.000000E+10  0.000000E+00  0.000000E+00  0.000000E+00  0.000000E+00  0.000000E+00  0.000000E+00  0.000000E+00  0.000000E+00  0.000000E+00  0.000000E+00  0.000000E+00  0.000000E+00       Funtag_D
      23  4.121232E+05  3.699735E+05       42149.7       19.6034     0.0149850      0.160304      0.191324      0.235683    0.00300436 -9.468820E-04    0.00806118       1.01927       Opttag_D
      23       77.5698       1.43745       1.00000  0.000000E+00       1.00000  0.000000E+00      0.884099       79.9271       1.00000       32.2526  0.000000E+00      0.880420  0.000000E+00      0.880420  0.000000E+00  0.000000E+00      -1.76084       79.3031       1.08731        1       Scatag_D
      23       1824.30       5661.40  0.000000E+00  0.000000E+00       2482.52  0.000000E+00       2716.20       7078.16  0.000000E+00       11767.1       60.9067       10559.2 -1.000000E+10 -1.000000E+10 -1.000000E+10 -1.000000E+10 -1.000000E+10  0.000000E+00  0.000000E+00  0.000000E+00  0.000000E+00  0.000000E+00  0.000000E+00  0.000000E+00  0.000000E+00  0.000000E+00  0.000000E+00  0.000000E+00  0.000000E+00       Funtag_D
      24  4.120691E+05  3.699229E+05       42146.3       18.8135     0.0157153      0.173879      0.191294      0.235678    0.00312709 -9.865662E-04    0.00806542       1.01930       Opttag_D
      24       77.6070       1.43679       1.00000  0.000000E+00       1.00000  0.000000E+00      0.884031       79.8799       1.00000       32.2707  0.000000E+00      0.872743  0.000000E+00      0.872743  0.000000E+00  0.000000E+00      -1.74549       79.3031       1.08731        1       Scatag_D
      24       1826.23       5661.70  0.000000E+00  0.000000E+00       2482.93  0.000000E+00       2716.33       7069.57  0.000000E+00       11768.4       60.8795       10560.2 -1.000000E+10 -1.000000E+10 -1.000000E+10 -1.000000E+10 -1.000000E+10  0.000000E+00  0.000000E+00  0.000000E+00  0.000000E+00  0.000000E+00  0.000000E+00  0.000000E+00  0.000000E+00  0.000000E+00  0.000000E+00  0.000000E+00  0.000000E+00       Funtag_D
      25  4.120216E+05  3.698981E+05       42123.4       13.5247     0.0159561      0.178897      0.191278      0.235665    0.00318711 -9.833418E-04    0.00806363       1.01878       Opttag_D
      25       77.6243       1.43665       1.00000  0.000000E+00       1.00000  0.000000E+00      0.884012       79.8707       1.00000       32.2773  0.000000E+00      0.869879  0.000000E+00      0.869879  0.000000E+00  0.000000E+00      -1.73976       79.3031       1.08731        1       Scatag_D
      25       1825.41       5655.96  0.000000E+00  0.000000E+00       2483.07  0.000000E+00       2716.82       7052.71  0.000000E+00       11769.5       60.8531       10559.1 -1.000000E+10 -1.000000E+10 -1.000000E+10 -1.000000E+10 -1.000000E+10  0.000000E+00  0.000000E+00  0.000000E+00  0.000000E+00  0.000000E+00  0.000000E+00  0.000000E+00  0.000000E+00  0.000000E+00  0.000000E+00  0.000000E+00  0.000000E+00       Funtag_D
      26  4.119523E+05  3.698353E+05       42117.0       13.1551     0.0168277      0.195962      0.191232      0.235645    0.00333957   -0.00100475    0.00806806       1.01881       Opttag_D
      26       77.6818       1.43662       1.00000  0.000000E+00       1.00000  0.000000E+00      0.883945       79.8371       1.00000       32.2997  0.000000E+00      0.860386  0.000000E+00      0.860386  0.000000E+00  0.000000E+00      -1.72077       79.3031       1.08731        1       Scatag_D
      26       1827.42       5656.23  0.000000E+00  0.000000E+00       2483.72  0.000000E+00       2718.83       7039.01  0.000000E+00       11773.3       60.8446       10557.7 -1.000000E+10 -1.000000E+10 -1.000000E+10 -1.000000E+10 -1.000000E+10  0.000000E+00  0.000000E+00  0.000000E+00  0.000000E+00  0.000000E+00  0.000000E+00  0.000000E+00  0.000000E+00  0.000000E+00  0.000000E+00  0.000000E+00  0.000000E+00       Funtag_D
      27  4.119217E+05  3.697916E+05       42130.1       19.8375     0.0177353      0.212893      0.191196      0.235642    0.00344556   -0.00106990    0.00807655       1.02009       Opttag_D
      27       77.7388       1.43716       1.00000  0.000000E+00       1.00000  0.000000E+00      0.883869       79.8079       1.00000       32.3222  0.000000E+00      0.851371  0.000000E+00      0.851371  0.000000E+00  0.000000E+00      -1.70274       79.3031       1.08731        1       Scatag_D
      27       1831.27       5670.53  0.000000E+00  0.000000E+00       2484.18  0.000000E+00       2721.02       7027.00  0.000000E+00       11777.1       60.8813       10558.1 -1.000000E+10 -1.000000E+10 -1.000000E+10 -1.000000E+10 -1.000000E+10  0.000000E+00  0.000000E+00  0.000000E+00  0.000000E+00  0.000000E+00  0.000000E+00  0.000000E+00  0.000000E+00  0.000000E+00  0.000000E+00  0.000000E+00  0.000000E+00       Funtag_D
      28  4.118844E+05  3.697823E+05       42102.1       12.1868     0.0179896      0.217709      0.191186      0.235644    0.00346823   -0.00109280    0.00807209       1.01900       Opttag_D
      28       77.7563       1.43752       1.00000  0.000000E+00       1.00000  0.000000E+00      0.883848       79.8035       1.00000       32.3278  0.000000E+00      0.848964  0.000000E+00      0.848964  0.000000E+00  0.000000E+00      -1.69793       79.3031       1.08731        1       Scatag_D
      28       1829.25       5658.40  0.000000E+00  0.000000E+00       2484.43  0.000000E+00       2721.65       7010.95  0.000000E+00       11778.6       60.9194       10557.9 -1.000000E+10 -1.000000E+10 -1.000000E+10 -1.000000E+10 -1.000000E+10  0.000000E+00  0.000000E+00  0.000000E+00  0.000000E+00  0.000000E+00  0.000000E+00  0.000000E+00  0.000000E+00  0.000000E+00  0.000000E+00  0.000000E+00  0.000000E+00       Funtag_D
      29  4.118299E+05  3.697542E+05       42075.7       9.47456     0.0188885      0.234389      0.191152      0.235648    0.00353637   -0.00117102    0.00807206       1.01879       Opttag_D
      29       77.8173       1.43897       1.00000  0.000000E+00       1.00000  0.000000E+00      0.883775       79.7905       1.00000       32.3475  0.000000E+00      0.840672  0.000000E+00      0.840672  0.000000E+00  0.000000E+00      -1.68134       79.3031       1.08731        1       Scatag_D
      29       1829.23       5656.06  0.000000E+00  0.000000E+00       2485.21  0.000000E+00       2723.91       6978.06  0.000000E+00       11784.0       61.0801       10558.1 -1.000000E+10 -1.000000E+10 -1.000000E+10 -1.000000E+10 -1.000000E+10  0.000000E+00  0.000000E+00  0.000000E+00  0.000000E+00  0.000000E+00  0.000000E+00  0.000000E+00  0.000000E+00  0.000000E+00  0.000000E+00  0.000000E+00  0.000000E+00       Funtag_D
      30  4.117982E+05  3.697339E+05       42064.3       10.5194     0.0195806      0.247014      0.191126      0.235645    0.00358566   -0.00121955    0.00807474       1.01892       Opttag_D
      30       77.8650       1.44021       1.00000  0.000000E+00       1.00000  0.000000E+00      0.883723       79.7841       1.00000       32.3623  0.000000E+00      0.834383  0.000000E+00      0.834383  0.000000E+00  0.000000E+00      -1.66877       79.3031       1.08731        1       Scatag_D
      30       1830.45       5657.46  0.000000E+00  0.000000E+00       2485.83  0.000000E+00       2725.49       6956.72  0.000000E+00       11788.6       61.2305       10558.5 -1.000000E+10 -1.000000E+10 -1.000000E+10 -1.000000E+10 -1.000000E+10  0.000000E+00  0.000000E+00  0.000000E+00  0.000000E+00  0.000000E+00  0.000000E+00  0.000000E+00  0.000000E+00  0.000000E+00  0.000000E+00  0.000000E+00  0.000000E+00       Funtag_D
      31  4.117573E+05  3.696888E+05       42068.5       21.8232     0.0216670      0.283844      0.191048      0.235627    0.00369497   -0.00135662    0.00808414       1.02077       Opttag_D
      31       78.0105       1.44472       1.00000  0.000000E+00       1.00000  0.000000E+00      0.883583       79.7780       1.00000       32.4055  0.000000E+00      0.816328  0.000000E+00      0.816328  0.000000E+00  0.000000E+00      -1.63266       79.3031       1.08731        1       Scatag_D
      31       1834.71       5678.03  0.000000E+00  0.000000E+00       2487.91  0.000000E+00       2729.91       6911.06  0.000000E+00       11804.4       61.7562       10560.7 -1.000000E+10 -1.000000E+10 -1.000000E+10 -1.000000E+10 -1.000000E+10  0.000000E+00  0.000000E+00  0.000000E+00  0.000000E+00  0.000000E+00  0.000000E+00  0.000000E+00  0.000000E+00  0.000000E+00  0.000000E+00  0.000000E+00  0.000000E+00       Funtag_D
      32  4.117089E+05  3.696766E+05       42032.3       10.0971     0.0220184      0.289906      0.191030      0.235613    0.00372463   -0.00135613    0.00807940       1.01909       Opttag_D
      32       78.0381       1.44563       1.00000  0.000000E+00       1.00000  0.000000E+00      0.883570       79.7816       1.00000       32.4112  0.000000E+00      0.813515  0.000000E+00      0.813515  0.000000E+00  0.000000E+00      -1.62703       79.3031       1.08731        1       Scatag_D
      32       1832.56       5659.43  0.000000E+00  0.000000E+00       2488.37  0.000000E+00       2729.81       6892.58  0.000000E+00       11807.5       61.8668       10560.2 -1.000000E+10 -1.000000E+10 -1.000000E+10 -1.000000E+10 -1.000000E+10  0.000000E+00  0.000000E+00  0.000000E+00  0.000000E+00  0.000000E+00  0.000000E+00  0.000000E+00  0.000000E+00  0.000000E+00  0.000000E+00  0.000000E+00  0.000000E+00       Funtag_D
      33  4.116846E+05  3.696666E+05       42018.0       9.30112     0.0220313      0.290156      0.191018      0.235600    0.00374885   -0.00132140    0.00807897       1.01908       Opttag_D
      33       78.0434       1.44578       1.00000  0.000000E+00       1.00000  0.000000E+00      0.883581       79.7873       1.00000       32.4087  0.000000E+00      0.813633  0.000000E+00      0.813633  0.000000E+00  0.000000E+00      -1.62727       79.3031       1.08731        1       Scatag_D
      33       1832.37       5659.23  0.000000E+00  0.000000E+00       2488.55  0.000000E+00       2728.67       6880.17  0.000000E+00       11808.4       61.8790       10558.7 -1.000000E+10 -1.000000E+10 -1.000000E+10 -1.000000E+10 -1.000000E+10  0.000000E+00  0.000000E+00  0.000000E+00  0.000000E+00  0.000000E+00  0.000000E+00  0.000000E+00  0.000000E+00  0.000000E+00  0.000000E+00  0.000000E+00  0.000000E+00       Funtag_D
      34  4.116411E+05  3.696326E+05       42008.5       11.1899     0.0226322      0.300272      0.190970      0.235555    0.00383154   -0.00125394    0.00808314       1.01932       Opttag_D
      34       78.0957       1.44747       1.00000  0.000000E+00       1.00000  0.000000E+00      0.883578       79.7993       1.00000       32.4141  0.000000E+00      0.809451  0.000000E+00      0.809451  0.000000E+00  0.000000E+00      -1.61890       79.3031       1.08731        1       Scatag_D
      34       1834.26       5661.94  0.000000E+00  0.000000E+00       2489.71  0.000000E+00       2726.91       6861.87  0.000000E+00       11815.0       61.9712       10556.8 -1.000000E+10 -1.000000E+10 -1.000000E+10 -1.000000E+10 -1.000000E+10  0.000000E+00  0.000000E+00  0.000000E+00  0.000000E+00  0.000000E+00  0.000000E+00  0.000000E+00  0.000000E+00  0.000000E+00  0.000000E+00  0.000000E+00  0.000000E+00       Funtag_D
      35  4.116320E+05  3.695792E+05       42052.8       27.0384     0.0241137      0.324239      0.190888      0.235486    0.00396105   -0.00116382    0.00810520       1.02230       Opttag_D
      35       78.2118       1.45152       1.00000  0.000000E+00       1.00000  0.000000E+00      0.883544       79.8171       1.00000       32.4339  0.000000E+00      0.799040  0.000000E+00      0.799040  0.000000E+00  0.000000E+00      -1.59808       79.3031       1.08731        1       Scatag_D
      35       1844.28       5695.07  0.000000E+00  0.000000E+00       2491.90  0.000000E+00       2725.86       6846.13  0.000000E+00       11830.8       62.0600       10556.7 -1.000000E+10 -1.000000E+10 -1.000000E+10 -1.000000E+10 -1.000000E+10  0.000000E+00  0.000000E+00  0.000000E+00  0.000000E+00  0.000000E+00  0.000000E+00  0.000000E+00  0.000000E+00  0.000000E+00  0.000000E+00  0.000000E+00  0.000000E+00       Funtag_D
      36  4.115833E+05  3.695754E+05       42007.9       10.1797     0.0241771      0.325246      0.190885      0.235483    0.00397030   -0.00115266    0.00809099       1.01953       Opttag_D
      36       78.2161       1.45156       1.00000  0.000000E+00       1.00000  0.000000E+00      0.883540       79.8148       1.00000       32.4355  0.000000E+00      0.798542  0.000000E+00      0.798542  0.000000E+00  0.000000E+00      -1.59708       79.3031       1.08731        1       Scatag_D
      36       1837.82       5664.22  0.000000E+00  0.000000E+00       2492.04  0.000000E+00       2725.77       6839.05  0.000000E+00       11831.0       62.0365       10556.0 -1.000000E+10 -1.000000E+10 -1.000000E+10 -1.000000E+10 -1.000000E+10  0.000000E+00  0.000000E+00  0.000000E+00  0.000000E+00  0.000000E+00  0.000000E+00  0.000000E+00  0.000000E+00  0.000000E+00  0.000000E+00  0.000000E+00  0.000000E+00       Funtag_D
      37  4.115623E+05  3.695605E+05       42001.8       6.79986     0.0246353      0.332436      0.190862      0.235467    0.00400631   -0.00112215    0.00809187       1.01948       Opttag_D
      37       78.2494       1.45263       1.00000  0.000000E+00       1.00000  0.000000E+00      0.883524       79.8152       1.00000       32.4434  0.000000E+00      0.795205  0.000000E+00      0.795205  0.000000E+00  0.000000E+00      -1.59041       79.3031       1.08731        1       Scatag_D
      37       1838.22       5663.69  0.000000E+00  0.000000E+00       2492.69  0.000000E+00       2725.73       6828.43  0.000000E+00       11835.1       62.0308       10555.9 -1.000000E+10 -1.000000E+10 -1.000000E+10 -1.000000E+10 -1.000000E+10  0.000000E+00  0.000000E+00  0.000000E+00  0.000000E+00  0.000000E+00  0.000000E+00  0.000000E+00  0.000000E+00  0.000000E+00  0.000000E+00  0.000000E+00  0.000000E+00       Funtag_D
      38  4.115375E+05  3.695288E+05       42008.6       8.29260     0.0256074      0.347230      0.190813      0.235425    0.00408321   -0.00103840    0.00809708       1.01985       Opttag_D
      38       78.3215       1.45507       1.00000  0.000000E+00       1.00000  0.000000E+00      0.883496       79.8196       1.00000       32.4593  0.000000E+00      0.788260  0.000000E+00      0.788260  0.000000E+00  0.000000E+00      -1.57652       79.3031       1.08731        1       Scatag_D
      38       1840.59       5667.83  0.000000E+00  0.000000E+00       2494.04  0.000000E+00       2725.39       6818.33  0.000000E+00       11844.7       62.0435       10555.7 -1.000000E+10 -1.000000E+10 -1.000000E+10 -1.000000E+10 -1.000000E+10  0.000000E+00  0.000000E+00  0.000000E+00  0.000000E+00  0.000000E+00  0.000000E+00  0.000000E+00  0.000000E+00  0.000000E+00  0.000000E+00  0.000000E+00  0.000000E+00       Funtag_D
      39  4.115063E+05  3.694800E+05       42026.3       12.4824     0.0269380      0.366002      0.190741      0.235352    0.00420177 -8.611475E-04    0.00810560       1.02040       Opttag_D
      39       78.4213       1.45843       1.00000  0.000000E+00       1.00000  0.000000E+00      0.883468       79.8271       1.00000       32.4802  0.000000E+00      0.778459  0.000000E+00      0.778459  0.000000E+00  0.000000E+00      -1.55692       79.3031       1.08731        1       Scatag_D
      39       1844.47       5673.88  0.000000E+00  0.000000E+00       2495.95  0.000000E+00       2724.37       6811.98  0.000000E+00       11858.6       62.1013       10555.0 -1.000000E+10 -1.000000E+10 -1.000000E+10 -1.000000E+10 -1.000000E+10  0.000000E+00  0.000000E+00  0.000000E+00  0.000000E+00  0.000000E+00  0.000000E+00  0.000000E+00  0.000000E+00  0.000000E+00  0.000000E+00  0.000000E+00  0.000000E+00       Funtag_D
      40  4.114764E+05  3.694159E+05       42060.4       11.1682     0.0288480      0.391436      0.190644      0.235242    0.00435719 -5.976609E-04    0.00811516       1.02060       Opttag_D
      40       78.5634       1.46340       1.00000  0.000000E+00       1.00000  0.000000E+00      0.883436       79.8386       1.00000       32.5125  0.000000E+00      0.764586  0.000000E+00      0.764586  0.000000E+00  0.000000E+00      -1.52917       79.3031       1.08731        1       Scatag_D
      40       1848.82       5676.14  0.000000E+00  0.000000E+00       2498.52  0.000000E+00       2723.11       6818.22  0.000000E+00       11879.1       62.1591       10554.4 -1.000000E+10 -1.000000E+10 -1.000000E+10 -1.000000E+10 -1.000000E+10  0.000000E+00  0.000000E+00  0.000000E+00  0.000000E+00  0.000000E+00  0.000000E+00  0.000000E+00  0.000000E+00  0.000000E+00  0.000000E+00  0.000000E+00  0.000000E+00       Funtag_D
      41  4.114507E+05  3.693942E+05       42056.5       7.30060     0.0291446      0.394040      0.190618      0.235210    0.00440994 -4.993597E-04    0.00811362       1.02029       Opttag_D
      41       78.5880       1.46408       1.00000  0.000000E+00       1.00000  0.000000E+00      0.883441       79.8414       1.00000       32.5171  0.000000E+00      0.761924  0.000000E+00      0.761924  0.000000E+00  0.000000E+00      -1.52385       79.3031       1.08731        1       Scatag_D
      41       1848.12       5672.67  0.000000E+00  0.000000E+00       2498.92  0.000000E+00       2722.32       6816.18  0.000000E+00       11882.8       62.1140       10553.3 -1.000000E+10 -1.000000E+10 -1.000000E+10 -1.000000E+10 -1.000000E+10  0.000000E+00  0.000000E+00  0.000000E+00  0.000000E+00  0.000000E+00  0.000000E+00  0.000000E+00  0.000000E+00  0.000000E+00  0.000000E+00  0.000000E+00  0.000000E+00       Funtag_D
      42  4.114235E+05  3.693627E+05       42060.7       9.21381     0.0293693      0.394427      0.190589      0.235170    0.00448628 -3.711301E-04    0.00811289       1.02031       Opttag_D
      42       78.6099       1.46447       1.00000  0.000000E+00       1.00000  0.000000E+00      0.883457       79.8436       1.00000       32.5205  0.000000E+00      0.759206  0.000000E+00      0.759206  0.000000E+00  0.000000E+00      -1.51841       79.3031       1.08731        1       Scatag_D
      42       1847.78       5672.92  0.000000E+00  0.000000E+00       2499.27  0.000000E+00       2721.13       6820.15  0.000000E+00       11885.8       62.0284       10551.7 -1.000000E+10 -1.000000E+10 -1.000000E+10 -1.000000E+10 -1.000000E+10  0.000000E+00  0.000000E+00  0.000000E+00  0.000000E+00  0.000000E+00  0.000000E+00  0.000000E+00  0.000000E+00  0.000000E+00  0.000000E+00  0.000000E+00  0.000000E+00       Funtag_D
      43  4.113950E+05  3.693104E+05       42084.6       11.3866     0.0301076      0.400045      0.190530      0.235101    0.00461331 -1.628707E-04    0.00811315       1.02023       Opttag_D
      43       78.6712       1.46623       1.00000  0.000000E+00       1.00000  0.000000E+00      0.883475       79.8494       1.00000       32.5345  0.000000E+00      0.752368  0.000000E+00      0.752368  0.000000E+00  0.000000E+00      -1.50474       79.3031       1.08731        1       Scatag_D
      43       1847.90       5671.99  0.000000E+00  0.000000E+00       2500.40  0.000000E+00       2719.52       6838.74  0.000000E+00       11894.3       61.8976       10549.8 -1.000000E+10 -1.000000E+10 -1.000000E+10 -1.000000E+10 -1.000000E+10  0.000000E+00  0.000000E+00  0.000000E+00  0.000000E+00  0.000000E+00  0.000000E+00  0.000000E+00  0.000000E+00  0.000000E+00  0.000000E+00  0.000000E+00  0.000000E+00       Funtag_D
      44  4.113660E+05  3.692600E+05       42106.0       10.3346     0.0309414      0.406132      0.190470      0.235034    0.00473562  2.215702E-05    0.00811229       1.02024       Opttag_D
      44       78.7392       1.46838       1.00000  0.000000E+00       1.00000  0.000000E+00      0.883494       79.8559       1.00000       32.5519  0.000000E+00      0.744876  0.000000E+00      0.744876  0.000000E+00  0.000000E+00      -1.48975       79.3031       1.08731        1       Scatag_D
      44       1847.51       5672.16  0.000000E+00  0.000000E+00       2501.60  0.000000E+00       2718.28       6852.84  0.000000E+00       11903.9       61.7490       10547.9 -1.000000E+10 -1.000000E+10 -1.000000E+10 -1.000000E+10 -1.000000E+10  0.000000E+00  0.000000E+00  0.000000E+00  0.000000E+00  0.000000E+00  0.000000E+00  0.000000E+00  0.000000E+00  0.000000E+00  0.000000E+00  0.000000E+00  0.000000E+00       Funtag_D
      45  4.113418E+05  3.692385E+05       42103.3       7.23069     0.0313838      0.409490      0.190442      0.235009    0.00478766  8.713993E-05    0.00811118       1.02019       Opttag_D
      45       78.7743       1.46958       1.00000  0.000000E+00       1.00000  0.000000E+00      0.883501       79.8586       1.00000       32.5621  0.000000E+00      0.741243  0.000000E+00      0.741243  0.000000E+00  0.000000E+00      -1.48249       79.3031       1.08731        1       Scatag_D
      45       1847.00       5671.65  0.000000E+00  0.000000E+00       2502.21  0.000000E+00       2717.82       6848.11  0.000000E+00       11908.5       61.6777       10546.3 -1.000000E+10 -1.000000E+10 -1.000000E+10 -1.000000E+10 -1.000000E+10  0.000000E+00  0.000000E+00  0.000000E+00  0.000000E+00  0.000000E+00  0.000000E+00  0.000000E+00  0.000000E+00  0.000000E+00  0.000000E+00  0.000000E+00  0.000000E+00       Funtag_D
      46  4.113161E+05  3.692087E+05       42107.4       7.41607     0.0320470      0.414210      0.190406      0.234977    0.00485997  1.665911E-04    0.00811042       1.02029       Opttag_D
      46       78.8260       1.47134       1.00000  0.000000E+00       1.00000  0.000000E+00      0.883510       79.8601       1.00000       32.5781  0.000000E+00      0.736002  0.000000E+00      0.736002  0.000000E+00  0.000000E+00      -1.47200       79.3031       1.08731        1       Scatag_D
      46       1846.66       5672.73  0.000000E+00  0.000000E+00       2503.19  0.000000E+00       2717.31       6847.27  0.000000E+00       11915.2       61.5781       10543.4 -1.000000E+10 -1.000000E+10 -1.000000E+10 -1.000000E+10 -1.000000E+10  0.000000E+00  0.000000E+00  0.000000E+00  0.000000E+00  0.000000E+00  0.000000E+00  0.000000E+00  0.000000E+00  0.000000E+00  0.000000E+00  0.000000E+00  0.000000E+00       Funtag_D
      47  4.112966E+05  3.691773E+05       42119.3       10.2782     0.0327118      0.417785      0.190369      0.234936    0.00493623  2.873799E-04    0.00811160       1.02039       Opttag_D
      47       78.8766       1.47300       1.00000  0.000000E+00       1.00000  0.000000E+00      0.883523       79.8600       1.00000       32.5934  0.000000E+00      0.730970  0.000000E+00      0.730970  0.000000E+00  0.000000E+00      -1.46194       79.3031       1.08731        1       Scatag_D
      47       1847.20       5673.84  0.000000E+00  0.000000E+00       2504.03  0.000000E+00       2717.26       6853.24  0.000000E+00       11921.8       61.5063       10540.4 -1.000000E+10 -1.000000E+10 -1.000000E+10 -1.000000E+10 -1.000000E+10  0.000000E+00  0.000000E+00  0.000000E+00  0.000000E+00  0.000000E+00  0.000000E+00  0.000000E+00  0.000000E+00  0.000000E+00  0.000000E+00  0.000000E+00  0.000000E+00       Funtag_D
      48  4.112724E+05  3.691486E+05       42123.8       7.97380     0.0333330      0.420516      0.190336      0.234896    0.00500593  3.930391E-04    0.00811211       1.02031       Opttag_D
      48       78.9227       1.47447       1.00000  0.000000E+00       1.00000  0.000000E+00      0.883536       79.8588       1.00000       32.6078  0.000000E+00      0.726398  0.000000E+00      0.726398  0.000000E+00  0.000000E+00      -1.45280       79.3031       1.08731        1       Scatag_D
      48       1847.43       5672.93  0.000000E+00  0.000000E+00       2504.93  0.000000E+00       2717.66       6854.33  0.000000E+00       11927.4       61.4260       10537.7 -1.000000E+10 -1.000000E+10 -1.000000E+10 -1.000000E+10 -1.000000E+10  0.000000E+00  0.000000E+00  0.000000E+00  0.000000E+00  0.000000E+00  0.000000E+00  0.000000E+00  0.000000E+00  0.000000E+00  0.000000E+00  0.000000E+00  0.000000E+00       Funtag_D
      49  4.112426E+05  3.690948E+05       42147.8       9.44434     0.0345292      0.424981      0.190274      0.234814    0.00513649  6.083260E-04    0.00811576       1.02052       Opttag_D
      49       79.0081       1.47717       1.00000  0.000000E+00       1.00000  0.000000E+00      0.883564       79.8552       1.00000       32.6355  0.000000E+00      0.717900  0.000000E+00      0.717900  0.000000E+00  0.000000E+00      -1.43580       79.3031       1.08731        1       Scatag_D
      49       1849.09       5675.29  0.000000E+00  0.000000E+00       2506.56  0.000000E+00       2719.28       6865.28  0.000000E+00       11937.8       61.2650       10533.3 -1.000000E+10 -1.000000E+10 -1.000000E+10 -1.000000E+10 -1.000000E+10  0.000000E+00  0.000000E+00  0.000000E+00  0.000000E+00  0.000000E+00  0.000000E+00  0.000000E+00  0.000000E+00  0.000000E+00  0.000000E+00  0.000000E+00  0.000000E+00       Funtag_D
      50  4.112237E+05  3.690591E+05       42164.6       10.6133     0.0354216      0.428422      0.190232      0.234763    0.00522300  7.294455E-04    0.00811838       1.02065       Opttag_D
      50       79.0692       1.47918       1.00000  0.000000E+00       1.00000  0.000000E+00      0.883583       79.8541       1.00000       32.6554  0.000000E+00      0.712042  0.000000E+00      0.712042  0.000000E+00  0.000000E+00      -1.42408       79.3031       1.08731        1       Scatag_D
      50       1850.29       5676.76  0.000000E+00  0.000000E+00       2507.71  0.000000E+00       2720.96       6872.18  0.000000E+00       11945.3       61.1746       10530.2 -1.000000E+10 -1.000000E+10 -1.000000E+10 -1.000000E+10 -1.000000E+10  0.000000E+00  0.000000E+00  0.000000E+00  0.000000E+00  0.000000E+00  0.000000E+00  0.000000E+00  0.000000E+00  0.000000E+00  0.000000E+00  0.000000E+00  0.000000E+00       Funtag_D
      51  4.112064E+05  3.690493E+05       42157.1       7.09329     0.0356240      0.429156      0.190221      0.234753    0.00524672  7.492050E-04    0.00811695       1.02045       Opttag_D
      51       79.0820       1.47954       1.00000  0.000000E+00       1.00000  0.000000E+00      0.883587       79.8532       1.00000       32.6591  0.000000E+00      0.710982  0.000000E+00      0.710982  0.000000E+00  0.000000E+00      -1.42196       79.3031       1.08731        1       Scatag_D
      51       1849.64       5674.50  0.000000E+00  0.000000E+00       2507.92  0.000000E+00       2721.52       6866.94  0.000000E+00       11946.3       61.1756       10529.1 -1.000000E+10 -1.000000E+10 -1.000000E+10 -1.000000E+10 -1.000000E+10  0.000000E+00  0.000000E+00  0.000000E+00  0.000000E+00  0.000000E+00  0.000000E+00  0.000000E+00  0.000000E+00  0.000000E+00  0.000000E+00  0.000000E+00  0.000000E+00       Funtag_D
      52  4.111812E+05  3.690243E+05       42156.9       6.38198     0.0362553      0.431988      0.190191      0.234729    0.00530745  7.887239E-04    0.00811724       1.02041       Opttag_D
      52       79.1214       1.48080       1.00000  0.000000E+00       1.00000  0.000000E+00      0.883600       79.8506       1.00000       32.6704  0.000000E+00      0.707845  0.000000E+00      0.707845  0.000000E+00  0.000000E+00      -1.41569       79.3031       1.08731        1       Scatag_D
      52       1849.77       5674.07  0.000000E+00  0.000000E+00       2508.58  0.000000E+00       2722.87       6863.80  0.000000E+00       11950.4       61.2300       10526.2 -1.000000E+10 -1.000000E+10 -1.000000E+10 -1.000000E+10 -1.000000E+10  0.000000E+00  0.000000E+00  0.000000E+00  0.000000E+00  0.000000E+00  0.000000E+00  0.000000E+00  0.000000E+00  0.000000E+00  0.000000E+00  0.000000E+00  0.000000E+00       Funtag_D
      53  4.111639E+05  3.690006E+05       42163.3       8.85538     0.0370801      0.436058      0.190156      0.234706    0.00536493  8.202232E-04    0.00811941       1.02059       Opttag_D
      53       79.1722       1.48264       1.00000  0.000000E+00       1.00000  0.000000E+00      0.883618       79.8460       1.00000       32.6851  0.000000E+00      0.703939  0.000000E+00      0.703939  0.000000E+00  0.000000E+00      -1.40788       79.3031       1.08731        1       Scatag_D
      53       1850.76       5676.01  0.000000E+00  0.000000E+00       2509.49  0.000000E+00       2723.78       6862.40  0.000000E+00       11956.8       61.3161       10522.7 -1.000000E+10 -1.000000E+10 -1.000000E+10 -1.000000E+10 -1.000000E+10  0.000000E+00  0.000000E+00  0.000000E+00  0.000000E+00  0.000000E+00  0.000000E+00  0.000000E+00  0.000000E+00  0.000000E+00  0.000000E+00  0.000000E+00  0.000000E+00       Funtag_D
      54  4.111397E+05  3.689730E+05       42166.7       7.39414     0.0383030      0.442393      0.190112      0.234679    0.00543209  8.362622E-04    0.00812236       1.02067       Opttag_D
      54       79.2443       1.48539       1.00000  0.000000E+00       1.00000  0.000000E+00      0.883643       79.8372       1.00000       32.7062  0.000000E+00      0.698770  0.000000E+00      0.698770  0.000000E+00  0.000000E+00      -1.39754       79.3031       1.08731        1       Scatag_D
      54       1852.10       5676.91  0.000000E+00  0.000000E+00       2510.82  0.000000E+00       2724.76       6856.48  0.000000E+00       11966.5       61.4457       10517.7 -1.000000E+10 -1.000000E+10 -1.000000E+10 -1.000000E+10 -1.000000E+10  0.000000E+00  0.000000E+00  0.000000E+00  0.000000E+00  0.000000E+00  0.000000E+00  0.000000E+00  0.000000E+00  0.000000E+00  0.000000E+00  0.000000E+00  0.000000E+00       Funtag_D
      55  4.111187E+05  3.689515E+05       42167.2       10.1323     0.0396789      0.449761      0.190072      0.234660    0.00548413  8.314671E-04    0.00812211       1.02100       Opttag_D
      55       79.3199       1.48841       1.00000  0.000000E+00       1.00000  0.000000E+00      0.883672       79.8200       1.00000       32.7272  0.000000E+00      0.693794  0.000000E+00      0.693794  0.000000E+00  0.000000E+00      -1.38759       79.3031       1.08731        1       Scatag_D
      55       1851.99       5680.62  0.000000E+00  0.000000E+00       2512.30  0.000000E+00       2724.63       6847.45  0.000000E+00       11977.5       61.5170       10511.2 -1.000000E+10 -1.000000E+10 -1.000000E+10 -1.000000E+10 -1.000000E+10  0.000000E+00  0.000000E+00  0.000000E+00  0.000000E+00  0.000000E+00  0.000000E+00  0.000000E+00  0.000000E+00  0.000000E+00  0.000000E+00  0.000000E+00  0.000000E+00       Funtag_D
      56  4.110981E+05  3.689408E+05       42157.3       6.61609     0.0405102      0.454382      0.190051      0.234653    0.00551005  8.039362E-04    0.00812051       1.02096       Opttag_D
      56       79.3621       1.49007       1.00000  0.000000E+00       1.00000  0.000000E+00      0.883690       79.8041       1.00000       32.7388  0.000000E+00      0.691334  0.000000E+00      0.691334  0.000000E+00  0.000000E+00      -1.38267       79.3031       1.08731        1       Scatag_D
      56       1851.26       5680.13  0.000000E+00  0.000000E+00       2513.20  0.000000E+00       2723.86       6837.18  0.000000E+00       11983.6       61.5302       10506.5 -1.000000E+10 -1.000000E+10 -1.000000E+10 -1.000000E+10 -1.000000E+10  0.000000E+00  0.000000E+00  0.000000E+00  0.000000E+00  0.000000E+00  0.000000E+00  0.000000E+00  0.000000E+00  0.000000E+00  0.000000E+00  0.000000E+00  0.000000E+00       Funtag_D
      57  4.110822E+05  3.689378E+05       42144.3       6.01810     0.0408878      0.456797      0.190043      0.234654    0.00551725  7.874837E-04    0.00811672       1.02097       Opttag_D
      57       79.3773       1.49061       1.00000  0.000000E+00       1.00000  0.000000E+00      0.883698       79.7896       1.00000       32.7416  0.000000E+00      0.690741  0.000000E+00      0.690741  0.000000E+00  0.000000E+00      -1.38148       79.3031       1.08731        1       Scatag_D
      57       1849.53       5680.29  0.000000E+00  0.000000E+00       2513.56  0.000000E+00       2722.57       6828.46  0.000000E+00       11985.4       61.4993       10503.1 -1.000000E+10 -1.000000E+10 -1.000000E+10 -1.000000E+10 -1.000000E+10  0.000000E+00  0.000000E+00  0.000000E+00  0.000000E+00  0.000000E+00  0.000000E+00  0.000000E+00  0.000000E+00  0.000000E+00  0.000000E+00  0.000000E+00  0.000000E+00       Funtag_D
      58  4.110620E+05  3.689329E+05       42129.2       8.44940     0.0416300      0.462149      0.190030      0.234658    0.00552932  7.681377E-04    0.00811080       1.02115       Opttag_D
      58       79.4053       1.49160       1.00000  0.000000E+00       1.00000  0.000000E+00      0.883712       79.7613       1.00000       32.7481  0.000000E+00      0.689434  0.000000E+00      0.689434  0.000000E+00  0.000000E+00      -1.37887       79.3031       1.08731        1       Scatag_D
      58       1846.83       5682.29  0.000000E+00  0.000000E+00       2514.27  0.000000E+00       2720.69       6818.83  0.000000E+00       11987.8       61.4936       10496.9 -1.000000E+10 -1.000000E+10 -1.000000E+10 -1.000000E+10 -1.000000E+10  0.000000E+00  0.000000E+00  0.000000E+00  0.000000E+00  0.000000E+00  0.000000E+00  0.000000E+00  0.000000E+00  0.000000E+00  0.000000E+00  0.000000E+00  0.000000E+00       Funtag_D
      59  4.110444E+05  3.689276E+05       42116.9       9.71056     0.0428248      0.488213      0.190009      0.234656    0.00554225  7.678897E-04    0.00810678       1.02147       Opttag_D
      59       79.4540       1.49354       1.00000  0.000000E+00       1.00000  0.000000E+00      0.883734       79.7306       1.00000       32.7633  0.000000E+00      0.686525  0.000000E+00      0.686525  0.000000E+00  0.000000E+00      -1.37305       79.3031       1.08731        1       Scatag_D
      59       1845.00       5685.82  0.000000E+00  0.000000E+00       2515.43  0.000000E+00       2720.04       6807.05  0.000000E+00       11992.3       61.5532       10489.7 -1.000000E+10 -1.000000E+10 -1.000000E+10 -1.000000E+10 -1.000000E+10  0.000000E+00  0.000000E+00  0.000000E+00  0.000000E+00  0.000000E+00  0.000000E+00  0.000000E+00  0.000000E+00  0.000000E+00  0.000000E+00  0.000000E+00  0.000000E+00       Funtag_D
      60  4.110279E+05  3.689242E+05       42103.6       6.04459     0.0431365      0.495864      0.190003      0.234654    0.00555030  7.902947E-04    0.00810554       1.02137       Opttag_D
      60       79.4657       1.49395       1.00000  0.000000E+00       1.00000  0.000000E+00      0.883739       79.7244       1.00000       32.7682  0.000000E+00      0.685574  0.000000E+00      0.685574  0.000000E+00  0.000000E+00      -1.37115       79.3031       1.08731        1       Scatag_D
      60       1844.44       5684.68  0.000000E+00  0.000000E+00       2515.66  0.000000E+00       2720.55       6796.65  0.000000E+00       11992.4       61.5879       10487.7 -1.000000E+10 -1.000000E+10 -1.000000E+10 -1.000000E+10 -1.000000E+10  0.000000E+00  0.000000E+00  0.000000E+00  0.000000E+00  0.000000E+00  0.000000E+00  0.000000E+00  0.000000E+00  0.000000E+00  0.000000E+00  0.000000E+00  0.000000E+00       Funtag_D
      61  4.110097E+05  3.689151E+05       42094.7       5.94487     0.0439580      0.514975      0.189984      0.234649    0.00557247  8.246877E-04    0.00810773       1.02137       Opttag_D
      61       79.4985       1.49515       1.00000  0.000000E+00       1.00000  0.000000E+00      0.883752       79.7109       1.00000       32.7818  0.000000E+00      0.683050  0.000000E+00      0.683050  0.000000E+00  0.000000E+00      -1.36610       79.3031       1.08731        1       Scatag_D
      61       1845.44       5684.78  0.000000E+00  0.000000E+00       2516.38  0.000000E+00       2721.97       6787.22  0.000000E+00       11993.7       61.6585       10483.5 -1.000000E+10 -1.000000E+10 -1.000000E+10 -1.000000E+10 -1.000000E+10  0.000000E+00  0.000000E+00  0.000000E+00  0.000000E+00  0.000000E+00  0.000000E+00  0.000000E+00  0.000000E+00  0.000000E+00  0.000000E+00  0.000000E+00  0.000000E+00       Funtag_D
      62  4.109949E+05  3.689044E+05       42090.4       7.53148     0.0449826      0.538947      0.189965      0.234644    0.00559831  8.538721E-04    0.00811167       1.02141       Opttag_D
      62       79.5370       1.49652       1.00000  0.000000E+00       1.00000  0.000000E+00      0.883768       79.6910       1.00000       32.7976  0.000000E+00      0.680305  0.000000E+00      0.680305  0.000000E+00  0.000000E+00      -1.36061       79.3031       1.08731        1       Scatag_D
      62       1847.23       5685.22  0.000000E+00  0.000000E+00       2517.29  0.000000E+00       2723.45       6781.72  0.000000E+00       11995.6       61.6989       10478.2 -1.000000E+10 -1.000000E+10 -1.000000E+10 -1.000000E+10 -1.000000E+10  0.000000E+00  0.000000E+00  0.000000E+00  0.000000E+00  0.000000E+00  0.000000E+00  0.000000E+00  0.000000E+00  0.000000E+00  0.000000E+00  0.000000E+00  0.000000E+00       Funtag_D
      63  4.109737E+05  3.688892E+05       42084.4       6.40576     0.0466639      0.577984      0.189937      0.234636    0.00563517  8.952098E-04    0.00812060       1.02151       Opttag_D
      63       79.5969       1.49862       1.00000  0.000000E+00       1.00000  0.000000E+00      0.883794       79.6569       1.00000       32.8237  0.000000E+00      0.676064  0.000000E+00      0.676064  0.000000E+00  0.000000E+00      -1.35213       79.3031       1.08731        1       Scatag_D
      63       1851.30       5686.33  0.000000E+00  0.000000E+00       2518.78  0.000000E+00       2726.10       6771.84  0.000000E+00       11998.2       61.7372       10470.1 -1.000000E+10 -1.000000E+10 -1.000000E+10 -1.000000E+10 -1.000000E+10  0.000000E+00  0.000000E+00  0.000000E+00  0.000000E+00  0.000000E+00  0.000000E+00  0.000000E+00  0.000000E+00  0.000000E+00  0.000000E+00  0.000000E+00  0.000000E+00       Funtag_D
      64  4.109559E+05  3.688742E+05       42081.7       7.78885     0.0478885      0.607131      0.189917      0.234633    0.00567175  9.210873E-04    0.00812790       1.02157       Opttag_D
      64       79.6343       1.49964       1.00000  0.000000E+00       1.00000  0.000000E+00      0.883811       79.6247       1.00000       32.8421  0.000000E+00      0.673338  0.000000E+00      0.673338  0.000000E+00  0.000000E+00      -1.34668       79.3031       1.08731        1       Scatag_D
      64       1854.63       5686.92  0.000000E+00  0.000000E+00       2519.91  0.000000E+00       2727.93       6767.69  0.000000E+00       11999.3       61.7426       10463.6 -1.000000E+10 -1.000000E+10 -1.000000E+10 -1.000000E+10 -1.000000E+10  0.000000E+00  0.000000E+00  0.000000E+00  0.000000E+00  0.000000E+00  0.000000E+00  0.000000E+00  0.000000E+00  0.000000E+00  0.000000E+00  0.000000E+00  0.000000E+00       Funtag_D
      65  4.109376E+05  3.688611E+05       42076.5       6.12642     0.0489327      0.632733      0.189903      0.234631    0.00570353  9.509331E-04    0.00813335       1.02158       Opttag_D
      65       79.6612       1.50016       1.00000  0.000000E+00       1.00000  0.000000E+00      0.883822       79.5911       1.00000       32.8573  0.000000E+00      0.671407  0.000000E+00      0.671407  0.000000E+00  0.000000E+00      -1.34281       79.3031       1.08731        1       Scatag_D
      65       1857.12       5687.05  0.000000E+00  0.000000E+00       2520.83  0.000000E+00       2729.17       6763.61  0.000000E+00       11999.6       61.7671       10457.3 -1.000000E+10 -1.000000E+10 -1.000000E+10 -1.000000E+10 -1.000000E+10  0.000000E+00  0.000000E+00  0.000000E+00  0.000000E+00  0.000000E+00  0.000000E+00  0.000000E+00  0.000000E+00  0.000000E+00  0.000000E+00  0.000000E+00  0.000000E+00       Funtag_D
      66  4.109234E+05  3.688526E+05       42070.8       6.04839     0.0494850      0.647432      0.189897      0.234636    0.00572408  9.693697E-04    0.00813479       1.02161       Opttag_D
      66       79.6696       1.49999       1.00000  0.000000E+00       1.00000  0.000000E+00      0.883823       79.5649       1.00000       32.8650  0.000000E+00      0.670701  0.000000E+00      0.670701  0.000000E+00  0.000000E+00      -1.34140       79.3031       1.08731        1       Scatag_D
      66       1857.78       5687.43  0.000000E+00  0.000000E+00       2521.31  0.000000E+00       2729.32       6760.85  0.000000E+00       11999.2       61.8332       10453.0 -1.000000E+10 -1.000000E+10 -1.000000E+10 -1.000000E+10 -1.000000E+10  0.000000E+00  0.000000E+00  0.000000E+00  0.000000E+00  0.000000E+00  0.000000E+00  0.000000E+00  0.000000E+00  0.000000E+00  0.000000E+00  0.000000E+00  0.000000E+00       Funtag_D
      67  4.109057E+05  3.688443E+05       42061.3       6.72896     0.0503582      0.671121      0.189896      0.234653    0.00574413  9.870622E-04    0.00813609       1.02166       Opttag_D
      67       79.6791       1.49960       1.00000  0.000000E+00       1.00000  0.000000E+00      0.883821       79.5196       1.00000       32.8769  0.000000E+00      0.669990  0.000000E+00      0.669990  0.000000E+00  0.000000E+00      -1.33998       79.3031       1.08731        1       Scatag_D
      67       1858.37       5687.97  0.000000E+00  0.000000E+00       2522.14  0.000000E+00       2729.09       6756.65  0.000000E+00       11999.0       61.9940       10446.1 -1.000000E+10 -1.000000E+10 -1.000000E+10 -1.000000E+10 -1.000000E+10  0.000000E+00  0.000000E+00  0.000000E+00  0.000000E+00  0.000000E+00  0.000000E+00  0.000000E+00  0.000000E+00  0.000000E+00  0.000000E+00  0.000000E+00  0.000000E+00       Funtag_D
      68  4.108890E+05  3.688446E+05       42044.4       7.97370     0.0516134      0.703082      0.189895      0.234675    0.00574350  9.983061E-04    0.00813770       1.02182       Opttag_D
      68       79.6990       1.49982       1.00000  0.000000E+00       1.00000  0.000000E+00      0.883817       79.4674       1.00000       32.8960  0.000000E+00      0.668734  0.000000E+00      0.668734  0.000000E+00  0.000000E+00      -1.33747       79.3031       1.08731        1       Scatag_D
      68       1859.10       5689.77  0.000000E+00  0.000000E+00       2523.29  0.000000E+00       2729.07       6743.10  0.000000E+00       12000.1       62.2084       10437.7 -1.000000E+10 -1.000000E+10 -1.000000E+10 -1.000000E+10 -1.000000E+10  0.000000E+00  0.000000E+00  0.000000E+00  0.000000E+00  0.000000E+00  0.000000E+00  0.000000E+00  0.000000E+00  0.000000E+00  0.000000E+00  0.000000E+00  0.000000E+00       Funtag_D
      69  4.108731E+05  3.688492E+05       42023.9       5.84299     0.0523210      0.720881      0.189899      0.234693    0.00573234  9.877237E-04    0.00813683       1.02181       Opttag_D
      69       79.7092       1.49999       1.00000  0.000000E+00       1.00000  0.000000E+00      0.883813       79.4382       1.00000       32.9068  0.000000E+00      0.668340  0.000000E+00      0.668340  0.000000E+00  0.000000E+00      -1.33668       79.3031       1.08731        1       Scatag_D
      69       1858.70       5689.68  0.000000E+00  0.000000E+00       2523.95  0.000000E+00       2728.74       6726.75  0.000000E+00       12000.8       62.3243       10432.9 -1.000000E+10 -1.000000E+10 -1.000000E+10 -1.000000E+10 -1.000000E+10  0.000000E+00  0.000000E+00  0.000000E+00  0.000000E+00  0.000000E+00  0.000000E+00  0.000000E+00  0.000000E+00  0.000000E+00  0.000000E+00  0.000000E+00  0.000000E+00       Funtag_D
      70  4.108574E+05  3.688564E+05       42000.9       5.91759     0.0535209      0.749975      0.189902      0.234719    0.00571483  9.611022E-04    0.00813686       1.02190       Opttag_D
      70       79.7289       1.50048       1.00000  0.000000E+00       1.00000  0.000000E+00      0.883806       79.3944       1.00000       32.9253  0.000000E+00      0.667687  0.000000E+00      0.667687  0.000000E+00  0.000000E+00      -1.33537       79.3031       1.08731        1       Scatag_D
      70       1858.72       5690.63  0.000000E+00  0.000000E+00       2525.08  0.000000E+00       2728.73       6707.79  0.000000E+00       12002.0       62.4306       10425.5 -1.000000E+10 -1.000000E+10 -1.000000E+10 -1.000000E+10 -1.000000E+10  0.000000E+00  0.000000E+00  0.000000E+00  0.000000E+00  0.000000E+00  0.000000E+00  0.000000E+00  0.000000E+00  0.000000E+00  0.000000E+00  0.000000E+00  0.000000E+00       Funtag_D
      71  4.108466E+05  3.688638E+05       41982.8       7.70888     0.0548164      0.780988      0.189906      0.234743    0.00569689  9.387798E-04    0.00813648       1.02207       Opttag_D
      71       79.7481       1.50086       1.00000  0.000000E+00       1.00000  0.000000E+00      0.883799       79.3460       1.00000       32.9448  0.000000E+00      0.667363  0.000000E+00      0.667363  0.000000E+00  0.000000E+00      -1.33473       79.3031       1.08731        1       Scatag_D
      71       1858.55       5692.56  0.000000E+00  0.000000E+00       2526.31  0.000000E+00       2728.98       6693.47  0.000000E+00       12002.7       62.4976       10417.7 -1.000000E+10 -1.000000E+10 -1.000000E+10 -1.000000E+10 -1.000000E+10  0.000000E+00  0.000000E+00  0.000000E+00  0.000000E+00  0.000000E+00  0.000000E+00  0.000000E+00  0.000000E+00  0.000000E+00  0.000000E+00  0.000000E+00  0.000000E+00       Funtag_D
      72  4.108341E+05  3.688658E+05       41968.3       5.29918     0.0556033      0.799645      0.189907      0.234755    0.00569202  9.167055E-04    0.00813605       1.02215       Opttag_D
      72       79.7583       1.50094       1.00000  0.000000E+00       1.00000  0.000000E+00      0.883793       79.3154       1.00000       32.9566  0.000000E+00      0.667416  0.000000E+00      0.667416  0.000000E+00  0.000000E+00      -1.33483       79.3031       1.08731        1       Scatag_D
      72       1858.35       5693.43  0.000000E+00  0.000000E+00       2527.05  0.000000E+00       2729.35       6682.06  0.000000E+00       12002.8       62.4885       10412.7 -1.000000E+10 -1.000000E+10 -1.000000E+10 -1.000000E+10 -1.000000E+10  0.000000E+00  0.000000E+00  0.000000E+00  0.000000E+00  0.000000E+00  0.000000E+00  0.000000E+00  0.000000E+00  0.000000E+00  0.000000E+00  0.000000E+00  0.000000E+00       Funtag_D
      73  4.108178E+05  3.688680E+05       41949.8       5.20310     0.0570080      0.832996      0.189913      0.234776    0.00568672  8.864463E-04    0.00813624       1.02232       Opttag_D
      73       79.7738       1.50087       1.00000  0.000000E+00       1.00000  0.000000E+00      0.883780       79.2595       1.00000       32.9776  0.000000E+00      0.667975  0.000000E+00      0.667975  0.000000E+00  0.000000E+00      -1.33595       79.3031       1.08731        1       Scatag_D
      73       1858.44       5695.34  0.000000E+00  0.000000E+00       2528.39  0.000000E+00       2730.23       6668.38  0.000000E+00       12002.9       62.4349       10403.7 -1.000000E+10 -1.000000E+10 -1.000000E+10 -1.000000E+10 -1.000000E+10  0.000000E+00  0.000000E+00  0.000000E+00  0.000000E+00  0.000000E+00  0.000000E+00  0.000000E+00  0.000000E+00  0.000000E+00  0.000000E+00  0.000000E+00  0.000000E+00       Funtag_D
      74  4.108062E+05  3.688740E+05       41932.2       8.14052     0.0586379      0.871718      0.189921      0.234804    0.00567232  8.589980E-04    0.00813608       1.02258       Opttag_D
      74       79.7903       1.50080       1.00000  0.000000E+00       1.00000  0.000000E+00      0.883765       79.1963       1.00000       33.0033  0.000000E+00      0.668709  0.000000E+00      0.668709  0.000000E+00  0.000000E+00      -1.33742       79.3031       1.08731        1       Scatag_D
      74       1858.36       5698.16  0.000000E+00  0.000000E+00       2530.02  0.000000E+00       2731.11       6655.23  0.000000E+00       12003.5       62.4540       10393.4 -1.000000E+10 -1.000000E+10 -1.000000E+10 -1.000000E+10 -1.000000E+10  0.000000E+00  0.000000E+00  0.000000E+00  0.000000E+00  0.000000E+00  0.000000E+00  0.000000E+00  0.000000E+00  0.000000E+00  0.000000E+00  0.000000E+00  0.000000E+00       Funtag_D
      75  4.107924E+05  3.688774E+05       41915.0       5.81724     0.0598211      0.899600      0.189927      0.234821    0.00566398  8.451085E-04    0.00813731       1.02267       Opttag_D
      75       79.8001       1.50058       1.00000  0.000000E+00       1.00000  0.000000E+00      0.883753       79.1499       1.00000       33.0223  0.000000E+00      0.669657  0.000000E+00      0.669657  0.000000E+00  0.000000E+00      -1.33931       79.3031       1.08731        1       Scatag_D
      75       1858.92       5699.18  0.000000E+00  0.000000E+00       2531.21  0.000000E+00       2731.78       6641.78  0.000000E+00       12004.0       62.4628       10385.7 -1.000000E+10 -1.000000E+10 -1.000000E+10 -1.000000E+10 -1.000000E+10  0.000000E+00  0.000000E+00  0.000000E+00  0.000000E+00  0.000000E+00  0.000000E+00  0.000000E+00  0.000000E+00  0.000000E+00  0.000000E+00  0.000000E+00  0.000000E+00       Funtag_D
      76  4.107765E+05  3.688814E+05       41895.1       5.81437     0.0613475      0.935163      0.189936      0.234843    0.00565410  8.456872E-04    0.00813847       1.02283       Opttag_D
      76       79.8100       1.50014       1.00000  0.000000E+00       1.00000  0.000000E+00      0.883732       79.0924       1.00000       33.0473  0.000000E+00      0.671240  0.000000E+00      0.671240  0.000000E+00  0.000000E+00      -1.34248       79.3031       1.08731        1       Scatag_D
      76       1859.46       5700.94  0.000000E+00  0.000000E+00       2532.79  0.000000E+00       2732.32       6627.16  0.000000E+00       12004.6       62.5383       10375.3 -1.000000E+10 -1.000000E+10 -1.000000E+10 -1.000000E+10 -1.000000E+10  0.000000E+00  0.000000E+00  0.000000E+00  0.000000E+00  0.000000E+00  0.000000E+00  0.000000E+00  0.000000E+00  0.000000E+00  0.000000E+00  0.000000E+00  0.000000E+00       Funtag_D
      77  4.107645E+05  3.688834E+05       41881.1       6.86141     0.0624032      0.959184      0.189943      0.234856    0.00564937  8.534588E-04    0.00813910       1.02291       Opttag_D
      77       79.8154       1.49973       1.00000  0.000000E+00       1.00000  0.000000E+00      0.883715       79.0559       1.00000       33.0645  0.000000E+00      0.672378  0.000000E+00      0.672378  0.000000E+00  0.000000E+00      -1.34476       79.3031       1.08731        1       Scatag_D
      77       1859.74       5701.90  0.000000E+00  0.000000E+00       2533.95  0.000000E+00       2732.39       6618.14  0.000000E+00       12004.6       62.6723       10367.7 -1.000000E+10 -1.000000E+10 -1.000000E+10 -1.000000E+10 -1.000000E+10  0.000000E+00  0.000000E+00  0.000000E+00  0.000000E+00  0.000000E+00  0.000000E+00  0.000000E+00  0.000000E+00  0.000000E+00  0.000000E+00  0.000000E+00  0.000000E+00       Funtag_D
      78  4.107517E+05  3.688825E+05       41869.2       6.21920     0.0630918      0.974330      0.189944      0.234859    0.00565156  8.696631E-04    0.00813877       1.02296       Opttag_D
      78       79.8176       1.49936       1.00000  0.000000E+00       1.00000  0.000000E+00      0.883700       79.0358       1.00000       33.0752  0.000000E+00      0.673190  0.000000E+00      0.673190  0.000000E+00  0.000000E+00      -1.34638       79.3031       1.08731        1       Scatag_D
      78       1859.59       5702.43  0.000000E+00  0.000000E+00       2534.73  0.000000E+00       2732.19       6610.88  0.000000E+00       12004.3       62.8061       10362.3 -1.000000E+10 -1.000000E+10 -1.000000E+10 -1.000000E+10 -1.000000E+10  0.000000E+00  0.000000E+00  0.000000E+00  0.000000E+00  0.000000E+00  0.000000E+00  0.000000E+00  0.000000E+00  0.000000E+00  0.000000E+00  0.000000E+00  0.000000E+00       Funtag_D
      79  4.107338E+05  3.688808E+05       41853.0       7.43644     0.0644812       1.00413      0.189950      0.234859    0.00565558  9.160441E-04    0.00813819       1.02313       Opttag_D
      79       79.8216       1.49871       1.00000  0.000000E+00       1.00000  0.000000E+00      0.883664       79.0041       1.00000       33.0951  0.000000E+00      0.674603  0.000000E+00      0.674603  0.000000E+00  0.000000E+00      -1.34921       79.3031       1.08731        1       Scatag_D
      79       1859.33       5704.32  0.000000E+00  0.000000E+00       2536.36  0.000000E+00       2731.90       6602.78  0.000000E+00       12003.6       63.0571       10351.7 -1.000000E+10 -1.000000E+10 -1.000000E+10 -1.000000E+10 -1.000000E+10  0.000000E+00  0.000000E+00  0.000000E+00  0.000000E+00  0.000000E+00  0.000000E+00  0.000000E+00  0.000000E+00  0.000000E+00  0.000000E+00  0.000000E+00  0.000000E+00       Funtag_D
      80  4.107217E+05  3.688811E+05       41840.6       7.87319     0.0655451       1.02669      0.189953      0.234857    0.00565509  9.283627E-04    0.00813862       1.02326       Opttag_D
      80       79.8263       1.49846       1.00000  0.000000E+00       1.00000  0.000000E+00      0.883640       78.9844       1.00000       33.1092  0.000000E+00      0.675161  0.000000E+00      0.675161  0.000000E+00  0.000000E+00      -1.35032       79.3031       1.08731        1       Scatag_D
      80       1859.52       5705.73  0.000000E+00  0.000000E+00       2537.71  0.000000E+00       2732.09       6594.52  0.000000E+00       12003.2       63.0958       10344.8 -1.000000E+10 -1.000000E+10 -1.000000E+10 -1.000000E+10 -1.000000E+10  0.000000E+00  0.000000E+00  0.000000E+00  0.000000E+00  0.000000E+00  0.000000E+00  0.000000E+00  0.000000E+00  0.000000E+00  0.000000E+00  0.000000E+00  0.000000E+00       Funtag_D
      81  4.107114E+05  3.688777E+05       41833.7       4.98723     0.0656140       1.02762      0.189951      0.234849    0.00566321  9.421695E-04    0.00813865       1.02316       Opttag_D
      81       79.8265       1.49844       1.00000  0.000000E+00       1.00000  0.000000E+00      0.883635       78.9895       1.00000       33.1083  0.000000E+00      0.674913  0.000000E+00      0.674913  0.000000E+00  0.000000E+00      -1.34983       79.3031       1.08731        1       Scatag_D
      81       1859.54       5704.62  0.000000E+00  0.000000E+00       2537.86  0.000000E+00       2732.02       6590.16  0.000000E+00       12002.7       63.0101       10343.8 -1.000000E+10 -1.000000E+10 -1.000000E+10 -1.000000E+10 -1.000000E+10  0.000000E+00  0.000000E+00  0.000000E+00  0.000000E+00  0.000000E+00  0.000000E+00  0.000000E+00  0.000000E+00  0.000000E+00  0.000000E+00  0.000000E+00  0.000000E+00       Funtag_D
      82  4.107004E+05  3.688735E+05       41826.9       4.22601     0.0660888       1.03711      0.189947      0.234835    0.00567352  9.724287E-04    0.00813941       1.02313       Opttag_D
      82       79.8288       1.49838       1.00000  0.000000E+00       1.00000  0.000000E+00      0.883620       78.9903       1.00000       33.1124  0.000000E+00      0.674611  0.000000E+00      0.674611  0.000000E+00  0.000000E+00      -1.34922       79.3031       1.08731        1       Scatag_D
      82       1859.88       5704.29  0.000000E+00  0.000000E+00       2538.60  0.000000E+00       2732.00       6586.81  0.000000E+00       12002.0       62.8585       10340.5 -1.000000E+10 -1.000000E+10 -1.000000E+10 -1.000000E+10 -1.000000E+10  0.000000E+00  0.000000E+00  0.000000E+00  0.000000E+00  0.000000E+00  0.000000E+00  0.000000E+00  0.000000E+00  0.000000E+00  0.000000E+00  0.000000E+00  0.000000E+00       Funtag_D
      83  4.106924E+05  3.688689E+05       41823.5       5.68842     0.0667853       1.05115      0.189945      0.234828    0.00568453    0.00101145    0.00814082       1.02314       Opttag_D
      83       79.8318       1.49819       1.00000  0.000000E+00       1.00000  0.000000E+00      0.883603       78.9842       1.00000       33.1209  0.000000E+00      0.674404  0.000000E+00      0.674404  0.000000E+00  0.000000E+00      -1.34881       79.3031       1.08731        1       Scatag_D
      83       1860.53       5704.46  0.000000E+00  0.000000E+00       2539.70  0.000000E+00       2731.84       6585.77  0.000000E+00       12001.7       62.7770       10336.7 -1.000000E+10 -1.000000E+10 -1.000000E+10 -1.000000E+10 -1.000000E+10  0.000000E+00  0.000000E+00  0.000000E+00  0.000000E+00  0.000000E+00  0.000000E+00  0.000000E+00  0.000000E+00  0.000000E+00  0.000000E+00  0.000000E+00  0.000000E+00       Funtag_D
      84  4.106816E+05  3.688615E+05       41820.1       5.26881     0.0677533       1.06993      0.189942      0.234816    0.00570247    0.00107255    0.00814316       1.02317       Opttag_D
      84       79.8358       1.49790       1.00000  0.000000E+00       1.00000  0.000000E+00      0.883579       78.9764       1.00000       33.1333  0.000000E+00      0.673993  0.000000E+00      0.673993  0.000000E+00  0.000000E+00      -1.34799       79.3031       1.08731        1       Scatag_D
      84       1861.60       5704.81  0.000000E+00  0.000000E+00       2541.29  0.000000E+00       2731.40       6583.95  0.000000E+00       12001.6       62.6780       10332.7 -1.000000E+10 -1.000000E+10 -1.000000E+10 -1.000000E+10 -1.000000E+10  0.000000E+00  0.000000E+00  0.000000E+00  0.000000E+00  0.000000E+00  0.000000E+00  0.000000E+00  0.000000E+00  0.000000E+00  0.000000E+00  0.000000E+00  0.000000E+00       Funtag_D
      85  4.106712E+05  3.688478E+05       41823.4       6.20764     0.0685157       1.08287      0.189934      0.234800    0.00573573    0.00115415    0.00814298       1.02325       Opttag_D
      85       79.8383       1.49749       1.00000  0.000000E+00       1.00000  0.000000E+00      0.883558       78.9749       1.00000       33.1424  0.000000E+00      0.673033  0.000000E+00      0.673033  0.000000E+00  0.000000E+00      -1.34607       79.3031       1.08731        1       Scatag_D
      85       1861.52       5705.68  0.000000E+00  0.000000E+00       2542.83  0.000000E+00       2730.30       6588.58  0.000000E+00       12001.4       62.6518       10330.4 -1.000000E+10 -1.000000E+10 -1.000000E+10 -1.000000E+10 -1.000000E+10  0.000000E+00  0.000000E+00  0.000000E+00  0.000000E+00  0.000000E+00  0.000000E+00  0.000000E+00  0.000000E+00  0.000000E+00  0.000000E+00  0.000000E+00  0.000000E+00       Funtag_D
      86  4.106604E+05  3.688323E+05       41828.1       4.35765     0.0690080       1.09006      0.189925      0.234784    0.00577344    0.00121913    0.00814341       1.02327       Opttag_D
      86       79.8395       1.49712       1.00000  0.000000E+00       1.00000  0.000000E+00      0.883542       78.9804       1.00000       33.1475  0.000000E+00      0.671729  0.000000E+00      0.671729  0.000000E+00  0.000000E+00      -1.34346       79.3031       1.08731        1       Scatag_D
      86       1861.71       5705.85  0.000000E+00  0.000000E+00       2544.05  0.000000E+00       2729.03       6594.22  0.000000E+00       12001.3       62.6994       10329.3 -1.000000E+10 -1.000000E+10 -1.000000E+10 -1.000000E+10 -1.000000E+10  0.000000E+00  0.000000E+00  0.000000E+00  0.000000E+00  0.000000E+00  0.000000E+00  0.000000E+00  0.000000E+00  0.000000E+00  0.000000E+00  0.000000E+00  0.000000E+00       Funtag_D
      87  4.106521E+05  3.688176E+05       41834.5       3.95119     0.0690758       1.08925      0.189917      0.234770    0.00580910    0.00127089    0.00814308       1.02323       Opttag_D
      87       79.8388       1.49683       1.00000  0.000000E+00       1.00000  0.000000E+00      0.883535       78.9914       1.00000       33.1464  0.000000E+00      0.670550  0.000000E+00      0.670550  0.000000E+00  0.000000E+00      -1.34110       79.3031       1.08731        1       Scatag_D
      87       1861.56       5705.44  0.000000E+00  0.000000E+00       2544.59  0.000000E+00       2727.80       6602.35  0.000000E+00       12001.2       62.8107       10328.8 -1.000000E+10 -1.000000E+10 -1.000000E+10 -1.000000E+10 -1.000000E+10  0.000000E+00  0.000000E+00  0.000000E+00  0.000000E+00  0.000000E+00  0.000000E+00  0.000000E+00  0.000000E+00  0.000000E+00  0.000000E+00  0.000000E+00  0.000000E+00       Funtag_D

   MaxLik Scaling Cycle 0093

   Wilson plot

   Statistics in resolution bins after refinement cycle 0003
```


|  |  |
| --- | --- |
|  | - Rxpct factors plot- Rxpct factors table- <Log-Lik Gain>- Ln Sigma A- Correlation Coefficients |


```
   Iters     Tot_Funct    Xray_Funct    Geom_Funct          Grms     rmsD2init     maxD2init             R         Rfree           LLG       LLGfree     rms(bond)    rms(angle)
   Iters             K   B_IMPF_FRAG        K_MISS   B_TEMP_MISS   K_IMPF_MISS   B_IMPF_MISS        K_SOLV   B_TEMP_SOLV   K_IMPF_SOLV   B_IMPF_SOLV        B_TEMP     B_TEMP_11     B_TEMP_12     B_TEMP_22     B_TEMP_13     B_TEMP_23     B_TEMP_33      K_WILSON      B_WILSON      FGCYCLES
   Iters          BOND         ANGLE      IMPROPER       TORSION    SINTORSION      TRIGONAL        PLANAR  BCORRELATION       CONTACT     IDEALDIST        CHIRAL    SIMILARITY       OCCSSUM  UTILDISTANCE     UTILANGLE   UTILTORSION        RHOFIT        ADP-01        ADP-02        ADP-03        ADP-04        ADP-05        ADP-06        ADP-07        ADP-08        ADP-09        ADP-10      RHOFITPC            QM
      88  4.106437E+05  3.687942E+05       41849.5       6.49669     0.0691918       1.08884      0.189900      0.234740    0.00586580    0.00135637    0.00814194       1.02321       Opttag_D
      88       79.8379       1.49651       1.00000  0.000000E+00       1.00000  0.000000E+00      0.883522       79.0176       1.00000       33.1426  0.000000E+00      0.668309  0.000000E+00      0.668309  0.000000E+00  0.000000E+00      -1.33662       79.3031       1.08731        1       Scatag_D
      88       1861.04       5705.27  0.000000E+00  0.000000E+00       2545.56  0.000000E+00       2725.91       6619.04  0.000000E+00       12001.2       62.9915       10328.4 -1.000000E+10 -1.000000E+10 -1.000000E+10 -1.000000E+10 -1.000000E+10  0.000000E+00  0.000000E+00  0.000000E+00  0.000000E+00  0.000000E+00  0.000000E+00  0.000000E+00  0.000000E+00  0.000000E+00  0.000000E+00  0.000000E+00  0.000000E+00       Funtag_D
   Geometry Restraint Deviations SCREEN Information

 
 No -bcorrel_zeroocchydrogens_factor flag found - keeping default: will set B for OCC=0 hydrogens to    1.200 times heavy-atom B


 TLS information. Total number of groups:     2


 Group   Natoms    FixRB   FixTLS   Group Tag    Group Spec
     1     2770      Y        Y     tls1    { A|* }
     2     2767      Y        Y     tls2    { B|* }
 
NOTE BUSTER_TLS_SET tls1 { A|* }
NOTE BUSTER_TLS_T   tls1     -1.71079E-01 -7.47635E-02  2.43417E-01 -2.86465E-02  4.33199E-02  1.72722E-03
NOTE BUSTER_TLS_L   tls1      1.08059E+00  8.73620E-01  2.76036E-01  2.69469E-01 -4.48190E-01  2.11414E-01
NOTE BUSTER_TLS_S   tls1      2.37944E-01 -2.31254E-01  1.94103E-01 -1.31455E-01 -6.52398E-02 -1.27204E-01 -5.47966E-02 -8.78254E-02
NOTE BUSTER_TLS_O   tls1     -2.33533E+01 -8.36748E+01  1.13031E+01
NOTE BUSTER_TLS_FIX tls1 ALL
 
NOTE BUSTER_TLS_SET tls2 { B|* }
NOTE BUSTER_TLS_T   tls2     -2.23653E-01 -1.74475E-01  3.03964E-01  1.65432E-02  5.01377E-02 -1.27065E-03
NOTE BUSTER_TLS_L   tls2      1.11201E+00  9.53119E-01  5.48649E-01  4.89954E-01 -5.70903E-01 -5.86900E-01
NOTE BUSTER_TLS_S   tls2     -3.19190E-02  1.25240E-01 -1.10983E-01  8.41507E-02  1.92000E-01  2.79219E-02 -6.18920E-02  9.91562E-02
NOTE BUSTER_TLS_O   tls2      2.20712E+01 -7.19054E+01  3.51200E+00
NOTE BUSTER_TLS_FIX tls2 ALL

 For final position, Geometry function summary
     term                           number     weight       rms        GooF      function     fn/numb
     ================================================================================================
     BOND bond lengths (angs)       5652       2.000      0.0081       0.406      1861.04       0.329
     ANGL bond angles (degs)        7669       2.000        1.02       0.610      5705.27       0.744
     TORS split-harmonic tors       1269       0.000       18.44       1.160         0.00       0.000
     SINTOR sinusoidal tors         1269       2.000       18.44                  2545.56       2.006
     PLAN general plane (angs)       963       5.000      0.0150       0.752      2725.91       2.831
     BCORrelations (angs^2)         5652      10.000       0.959       0.342      6619.04       1.171
     CONT (bad contacts)               0       5.000                                 0.00
     IDEAL(ideal-dist contact)      5538       4.000                             12001.24
     CHIRAL (gelly semiharmon)       740       5.000                                62.99
     SIM similarity restraint for NCS and/or TARGET                              10328.42
     OCCSUM restraint on sum of occupanies                                           0.00       as none defined
     DISTAN utility restraints interatomic distance                                  0.00       as none defined
     UTILANGLE utility angle restraints                                              0.00       as none defined
     UTILTOR utility restraints on torsion angles                                    0.00       as none defined
     Peptide omega torsion angles                           3.04 degs - monitor only

 Weighted rms      0.0081         1.023  (temporary rms bond and angle for BUSTER to pick up!)
 Weighted intra-residual rms(bond) for StdProtein (excluding H)      0.0079
 Weighted intra-residual rms(bond) for Buffer     (excluding H)      0.0030
 Outputing geometry progress graph in plotmtv format to file:Geom_Res.mtv

 ############### Running final geometry/screen analysis (screen_final.txt)

 s/r gelly normal termination
```

Statistics and scale factors vs. cycle number
```

|  |  |  |
| --- | --- | --- |
|  | - Rxpct factors- <Log-Lik Gain>- GEOM Residuals- Ln Sigma_A_ | - Scale Factors- B Factors- Imperfection Factors |

```
   Final Geometry Information


---


### 2. Final results

Statistics and scale factors vs. cycle number
```

|  |  |  |
| --- | --- | --- |
|  | - Rxpct factors- <Log-Lik Gain>- GEOM Residuals- Ln Sigma_A_ | - Scale Factors- B Factors- Imperfection Factors |

```
   Average Figure of Merit
   Structure Factor Amplitudes


 # normal termination

  Program stopped.
```

closesok message: time spent in tree = 1435.76 (user) 28.61 (sys)

---

# Normal termination

```
 Resources usage summary:
     Time spent in client ...................  1435.76 (user)       28.64 (system)
     Time spent in scripts (    27 calls) ...     2.37 (user)        0.17 (system)
     Time spent in server ...................     0.00 (user)        0.01 (system)

     Max resident memory used (kb) .......... 415884
     Number of page faults .................. 0 (major)    360592 (minor)
     Number of context switches ............. 2309379 (niced)    6598 (other)
```
